# Facile fabrication of luminescent organic dots by thermolysis of citric acid in urea melt, and their use for cell staining and polyelectrolyte microcapsule labelling

**DOI:** 10.3762/bjnano.7.182

**Published:** 2016-12-02

**Authors:** Nadezhda M Zholobak, Anton L Popov, Alexander B Shcherbakov, Nelly R Popova, Mykhailo M Guzyk, Valeriy P Antonovich, Alla V Yegorova, Yuliya V Scrypynets, Inna I Leonenko, Alexander Ye Baranchikov, Vladimir K Ivanov

**Affiliations:** 1 Zabolotny Institute of Microbiology and Virology, National Academy of Sciences of Ukraine, Kyiv 03680, Ukraine; 2 Institute of Theoretical and Experimental Biophysics, Pushchino 142290, Russia; 3 Palladin Institute of Biochemistry NAS of Ukraine, Kyiv 01601, Ukraine; 4 Bogatsky Physico-Chemical Institute, National Academy of Sciences of Ukraine, Odessa 65080, Ukraine; 5 Kurnakov Institute of General and Inorganic Chemistry of the Russian Academy of Sciences, Moscow 119991, Russia; 6 National Research Tomsk State University, Tomsk 634050, Russia

**Keywords:** cell culture, citric acid, layer-by-layer (LbL)-microcapsules, luminescence, organic dots (O-dots), staining, toxicity

## Abstract

Luminescent organic dots (O-dots) were synthesized via a one-pot, solvent-free thermolysis of citric acid in urea melt. The influence of the ratio of the precursors and the duration of the process on the properties of the O-dots was established and a mechanism of their formation was hypothesized. The multicolour luminescence tunability and toxicity of synthesized O-dots were extensively studied. The possible applications of O-dots for alive/fixed cell staining and labelling of layer-by-layer polyelectrolyte microcapsules were evaluated.

## Introduction

Luminescent nanosized semiconductor crystals (quantum dots, Q-dots) are a good alternative to common fluorescent dyes in a variety of biomedical applications, mainly due to their high photostability and relatively large Stokes shift [1–3], but Q-dots typically contain heavy metals (lead, cadmium) and chalcogens (selenium, tellurium), making them quite toxic. In turn, nanophosphors based on rare earth elements (REE) are less harmful, but very expensive.

Today, scientists are exploring the possibility of using nanoscale fluorescent carbon dots (C-dots) instead of Q-dots and REE-based nanophosphors. C-dots possess the attractive properties of low toxicity, being environmentally friendly, offering simple synthetic routes and low cost, as well as having comparable optical properties to traditional quantum dots and organic dyes [4–18]. Photoluminescent C-dots are superior in terms of solubility, outstanding biocompatibility, resistance to photobleaching, chemical inertness and excellent suitability for biological applications (one- and multiphoton bioimaging [9–16], biosensorics [9–12,17] and biomolecules/drug delivery [12–13,15]). Since the discovery of luminescent C-dots in 2004 [18], the number of annual peer-reviewed publications on the biomedical applications of C-dots dramatically increases – especially in comparison with the relatively smooth growth of publications on other fluorescent nanoparticles (for instance, REE-based).

Typically, С-dots are not made only of carbon, but also contain other elements, including hydrogen, oxygen and nitrogen. Fluorescent sites of C-dots are usually organic molecules bound by intermolecular or covalent forces. Therefore, these nanostructures should, more precisely, be called organic dots (O-dots), or luminescent organic clusters (LOC). A semiconductor quantum dot is a single collective electronic oscillator; in contrast, an organic dot is probably a clustered pack of isolated oscillators (phosphors) [19]. According to literature data, depending on the reaction conditions, (temperature, duration of the process and the ratio of precursors, among others), the O-dots conditionally fall into two main categories. The first type of O-dots is formed under mild conditions, e.g., at relatively low temperatures. In this case, each dot consists of one kind of phosphor, joined in a particle mainly due to weak (physical) forces; with further heating, type-I dots can be transformed into type-II dots, but not vice versa (see Supporting Information File 1, Figure S1). Organic type-II dots are usually the products of deep carbonization of pristine organic substances, and are more like carbon. The structure of type-II O-dots consists of different oscillators joined together by stronger bonds, (for example, by σ-bonds between carbon atoms). Due to the presence of multiple independent oscillators, the absorption spectra of type-II O-dots do not consist of individual bands, (in contrast to type-I O-dots). Another distinctive feature of type-II dots is that their emission wavelength depends on the excitation wavelength. In contrast, type-I O-dots obey Kasha's rule; the excitation wavelength affects the intensity of the luminescence only, but not the wavelength (colour) of the emitted light (see Supporting Information File 1, Figure S2 and Figure S3). There are also intermediate types of O-dots: for example, during the formation of O-dots by the joining of separate harmonic oscillators (molecules), transition states can form when the system has two or more modes of excitation and emission (see Supporting Information File 1, Figure S4).

Among the numerous families of O-dots, the most popular nanostructures are prepared by a “bottom up” route, via the thermolysis of various organic compounds. For example, when heated citric acid and its salts are transformed easily into O-dots [20–25] (see Supporting Information File 1, Figure S1). Thus, the hydrothermal treatment of an equimolar mixture of citric acid and sodium hydroxide solutions in an autoclave at 160 °C for 4 h leads to the formation of O-dots having a quantum yield of about 22% [21]. Similarly, hydrothermal treatment of a solution of sodium citrate and ammonium bicarbonate at 180 °C for 4 h leads to the formation of O-dots with excellent photostability [22]. When a 0.328 M solution of ammonium citrate was subjected to hydrothermal treatment in an autoclave at 160 °C for 6 h, the resulting dots had a quantum yield of about 13.5% [23]. Bourlinos et al. [24–25] heated various citrate salts, e.g., ammonium citrate in air, up to 300 °C, to produce water-soluble carbon dots of 7 nm average diameter and a fluorescence quantum yield of 3% at 495 nm excitation [24].

Doping of citric acid-based organic dots with nitrogen greatly improves their luminescent properties. A large number of studies have been devoted to the synthesis of citrate O-dots using urea as a nitrogen source [21,24,26–30]. For instance, highly luminescent O-dots were obtained by hydrothermal treatment of citric acid and urea or thiourea (molar ratio 1:3) solutions, in an autoclave at 160 °C for 4 h [26]. Qu et al. [27–28] synthesized luminescent dots, using a microwave-hydrothermal method, from urea and citric acid, using mass ratios of 0.2:1, 2:1 [27] or 1:1 [28]. Citric acid and urea were added to distilled water to form a transparent solution. The solution was then heated in a domestic 650 W microwave oven for 4–5 min, during which the solution changed from a colourless liquid to brown, and finally to a dark-brown, clustered solid. Hou et al. [29] proposed a simple, low-cost, one-pot method to synthesize water-soluble, fluorescent dots through electrochemical carbonization of sodium citrate and urea. Citric acid- and urea-based organic dots were also synthesized by microwave heating at 180 °C, in a solution of oleic acid [30]. It should be noted that all the above-mentioned syntheses were carried out in solutions, (aqueous or non-aqueous), and required special, sophisticated equipment, such as autoclaves and/or microwave setups.

In this paper, we have focused our efforts on the development of a new procedure for the solvent-free synthesis of O-dots via thermolysis of citric acid in urea melt. In this procedure, facile tuning of optical characteristics and cellular toxicity is possible simply by changing the processing parameters, e.g., the ratio of precursors or the duration of synthesis. We have succeeded in the decoration of polyelectrolyte microcapsules with O-dots synthesized in this way, the former being considered very attractive drug-delivery vehicles in living beings [31–32]. Earlier, such microcapsules had been decorated using other luminescent labels, including organic dyes [33], rare-earth phosphate nanocrystals [34] and visible [35–36] or near-infrared emitting chalcogenide Q-dots [36].

## Results and Discussion

### O-dot formation and spectral properties

**Figure 1:**
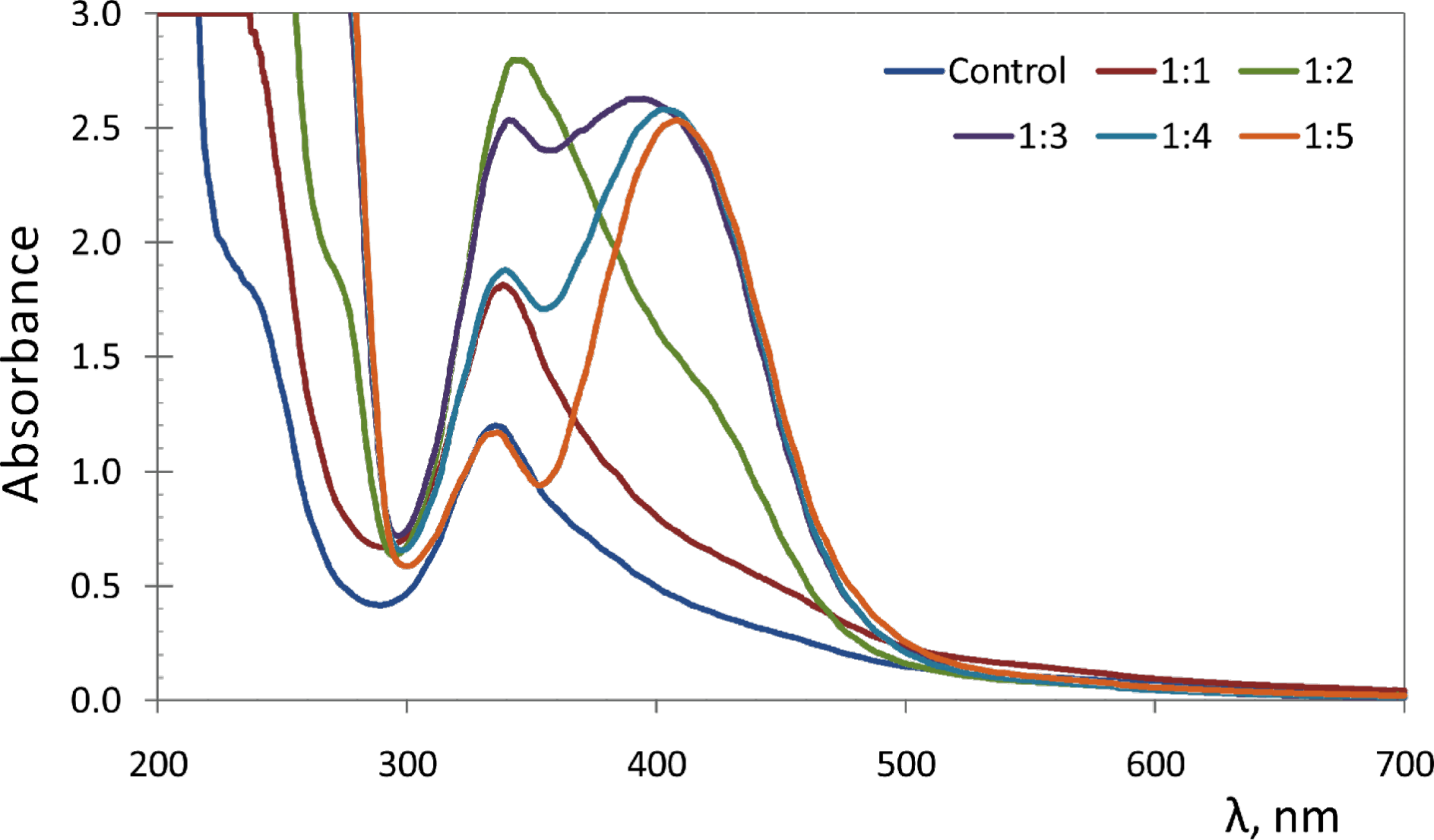
Absorption spectra of citric acid heated in the urea melt (1:1, 1:2, 1:3, 1:4 and 1:5 molar ratios) at 160 °C for 120 min (with subsequent dissolution of the reaction products in water). The control sample consisted of the product of thermal treatment of triammonium citrate (without urea) under the same conditions.

**Figure 2:**
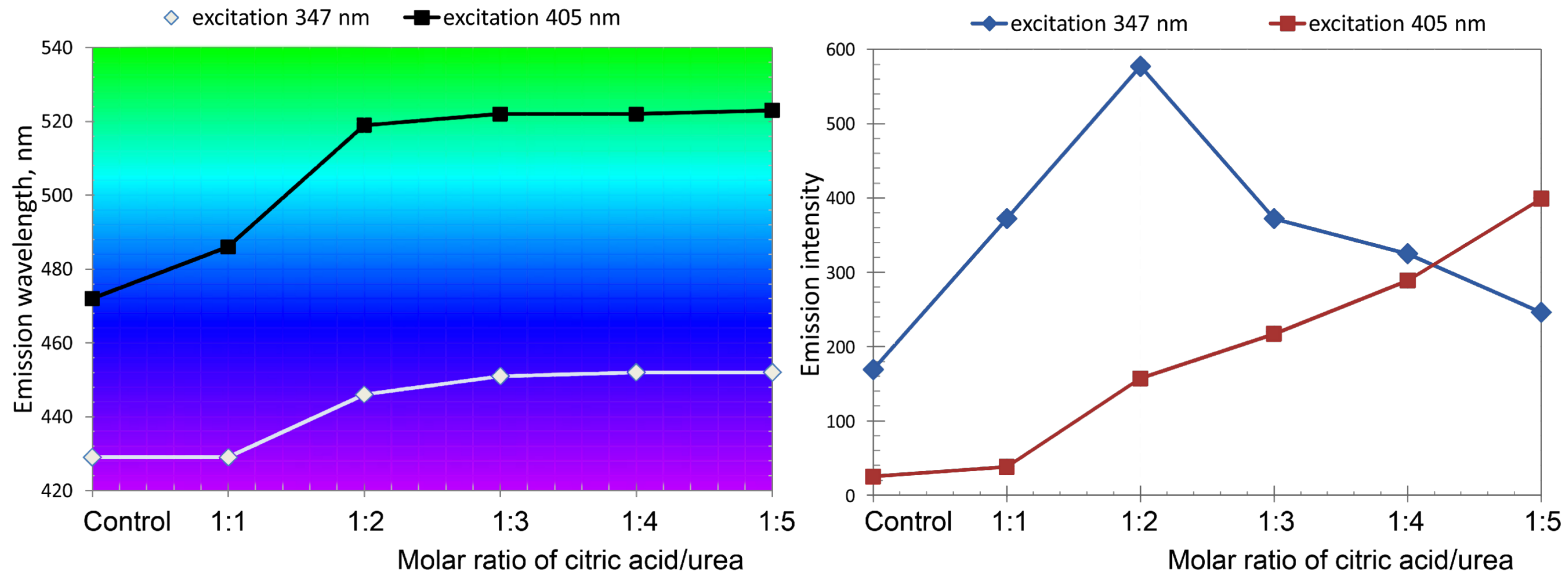
Left: Maximum emission wavelengths of the products obtained by heating citric acid in the urea melt at 160 °C for 120 min (with subsequent dissolution in water). The colour of the graph areas corresponds to the colour of luminescence. Right: Luminescence intensity of the citric acid heated in the urea melt at 160 °C for 120 min (aqueous solution 2 μg/mL). The *x*-axis is molar ratio of citric acid to urea. The control sample consisted of the products of thermal treatment of triammonium citrate (without urea) under the same conditions.

Citric acid reacts with ammonia in a molar ratio of 1:3 to form triammonium citrate, and with urea in a molar ratio of 1:1 [37] to form urea citrate. Thermolysis of ammonium citrate or urea citrate leads to formation of the luminescent O-dots, proceeding readily and at moderate temperatures, in the excess of urea melt, (melting point 133 °C). The duration of the process strongly depends on the temperature. The optimum temperature range was found to be 150–180 °C. In this temperature range, the process of O-dot formation was completed in 1–2 h. Upon changing the molar ratio of citric acid/urea in the precursor mixtures from 1:1 to 1:2, 1:3, 1:4 and 1:5, the absorption spectra of products were changed (Figure 1) and the luminescence emission maxima were shifted to higher wavelengths (Figure 2, left). The dependence of luminescence intensity from the citric acid/urea molar ratio was non-linear (Figure 2, right). More details on the absorption, excitation and emission spectra of the samples are presented in Supporting Information File 1, Figures S5–S10.

Upon heating, decomposition and release of gaseous products such as water, ammonia, carbon dioxide took place. Useful indicators of process completion are the stabilization of the system weight and carbonization (hardening) of the melt. At a temperature of 160 °C, the mixture hardened in 1.5–2.0 hours, and after that its weight varied only slightly (see Supporting Information File 1, Figure S11).

**Figure 3:**
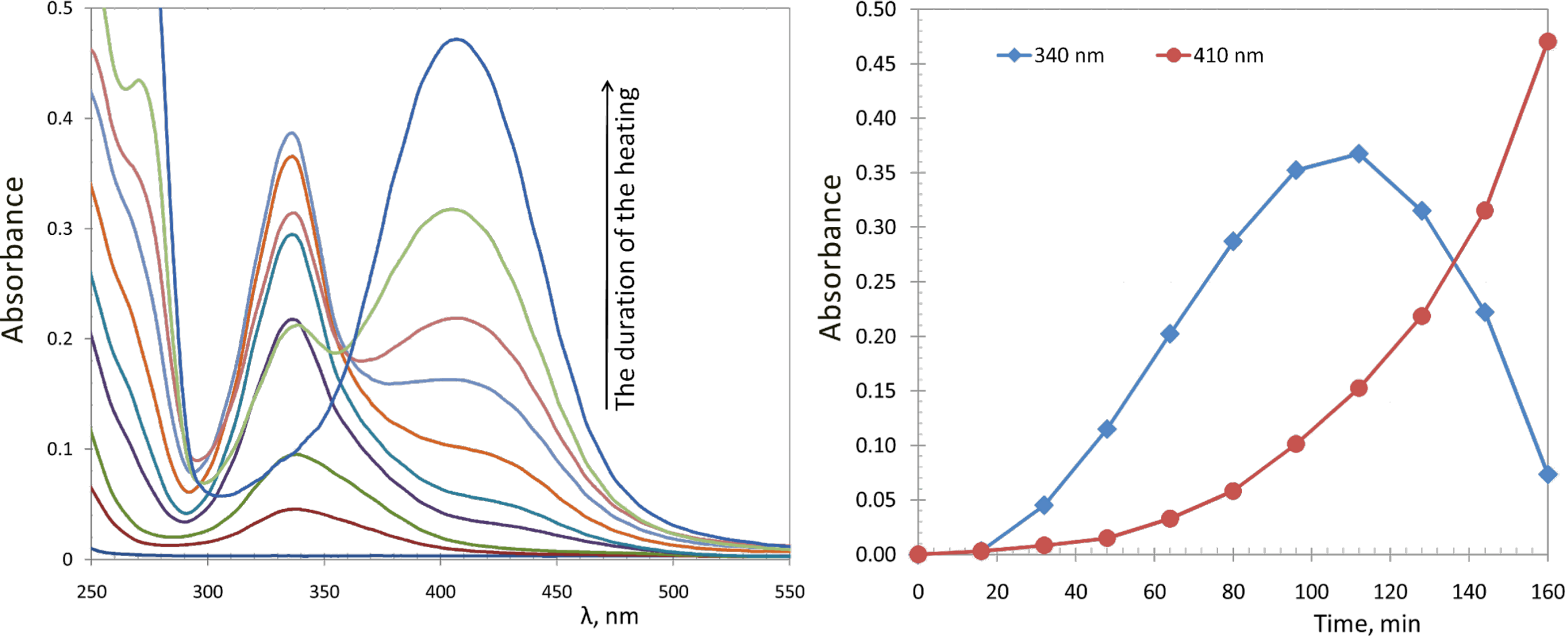
Left: The changes in the absorption spectra of a mixture of citric acid and urea (1:5 mol) upon heating at 160 °C for 0–160 min (with subsequent dissolution of the reaction products in water). Right: Sequential changes in optical density at the absorption maxima of 340 nm and 410 nm.

The absorption spectrum of the mixture changed in the course of heating: At the beginning of the process, the absorption band at about 340 nm appeared, increased and then decreased and disappeared (Figure 3, left); simultaneously, a new band at about 410 nm appeared and began to increase monotonically (Figure 3, right). One can assume that condensation occurs sequentially, starting from the formation of some units based on the precursor ratio 1:1, and proceeding through the merging of these units by means of the products of urea thermolysis.

The observed results are consistent with the mechanism of the formation of O-dots by combining fluorophores formed via the condensation of the molecules of citric acid and amines [38–40]. According to the suggested reaction mechanism, ethylenediamine and citric acid form a salt at elevated temperatures, before reacting to a large network, due to the multiple functional groups of both precursor molecules. It was found that, aside from the polymerization reaction, a salt of citric acid and ethylenediamine forms the fluorescent molecule 5-oxo-1,2,3,5-tetrahydroimidazo[1,2-α]pyridine-77-carboxylic acid (IPCA) in a second simultaneous intramolecular reaction. The subsequent rise in temperature (up to 200 °C) causes the formation of polymerized species forming amorphous carbon dots with partial sp^2^ hybridization. Song et al. demonstrated [39] that the primary fluorophore of a carbon dot is an independent fluorescent molecule, or possibly a molecule linked to the surface or incorporated inside the carbon core. It is well known that IPCA is the derivative of citrazinic (2,6-dihydroxypyridine-4-carboxylic) acid. In our experiments, the primary fluorophore is probably the pristine citrazinic acid in the form of ammonium salt or amide, which is readily formed by thermolysis of the citric acid and ammonia [41–42] released in the course of urea decomposition. It was found that the heating of pure ammonium citrazinate at 160 °C for 120 min did not affect its absorption and emission spectra. Conversely, heating ammonium citrazinate in the urea melt led to O-dots formation (Supporting Information File 1, Figure S12). Note that the heating of triammonium citrate under the same conditions produced a product that also had a bluish luminescence; the position of the absorption and emission bands were nearly independent from the duration of the heating process (see Supporting Information File 1, Figure S13). When the product obtained by thermal treatment of triammonium citrate was further heated in the urea melt, O-dots having a bright-green luminescence were formed. Depending on the pH value, pure citrazinic acid has a characteristic UV absorption band at 340–345 nm [43] and a pronounced blue luminescence. Its heating in the urea melt was accompanied by the condensation of fluorophores, which caused a bathochromic shift of the absorption, excitation and emission bands (Supporting Information File 1, Figures S4 and S12), wherein quantum yield varied non-linearly in the range of 7–16% (see Supporting Information File 1, Figure S14), with the emission intensity of the samples (Figure 2, right). During the formation of the O-dots, the distance between fluorophores decreases, and this should lead to some autoquenching. Earlier, a similar bathochromic shift in the emission spectra of monomeric fluorophores upon condensation in a polymer matrix was registered by Ito et al. for perylene [44], a dibenzoylmethane boron complex [45] and a cyanostilbene derivative [46]. The latter paper also reported that the quantum yield of monomer fluorescence was higher than that of the aggregates.

**Scheme 1:**
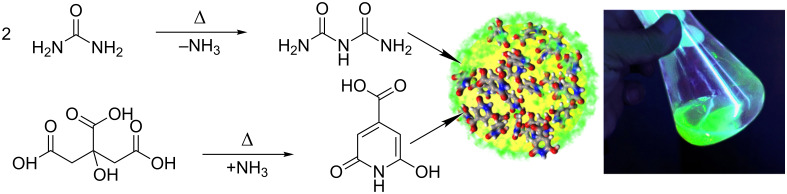
Left: Possible mechanism of O-dot formation. Right: Appearance of 0.01% O-dots colloidal solution under UV illumination.

A possible mechanism of O-dot formation is represented in Scheme 1, which also demonstrates the appearance of 0.01% O-dots solution under UV illumination.

The formation of a fluorescent O-dot requires the presence not only of primary fluorophores, but also of a binder joining them together, for instance, the products of urea thermolysis. Thus, the resulting O-dot is a set of primary fluorophores bound by covalent or intermolecular (coordination, donor–acceptor, hydrogen) bonds, for example, forming a polymer-like structure [47], so that the particles formed can be identified as clusters of independent fluorophores [19]. Solubilization of these clusters in water leads to micellar peptization and colloidal sol formation; peptization increases with temperature and dilution. The colloidal nature of the final O-dots solution can be demonstrated easily by the Tyndall cone arising at wavelengths where absorption is absent, for example, when illuminated with a red laser pointer (635 nm). The digital photograph (Supporting Information File 1, Figure S15, insert) shows a notable Tyndall effect in the colloidal solution of O-dots (right), whereas the true molecular solution of fluorescein having the same concentration (100 μg/mL) did not scatter the red laser beam (left). Dynamic light scattering (DLS) measurements indicated that the O-dots tended to agglomerate in the solution, and the size of the agglomerates increased with increasing concentration (Supporting Information File 1, Figure S15). The agglomerates had a relatively high polydispersity index, (in the 100 μg/mL colloidal solution of O-dots, the hydrodynamic diameter was about 300 ± 200 nm), and negative zeta-potential (in the range of pH 5.0…9.0, ζ = −31…−35 mV, see Supporting Information File 1, Figure S16). Such behaviour resembles the properties of surfactants that are prone to micelle formation. On the other hand, the observed behaviour of O-dots is very convenient for their use in decorating the surface of microcapsules.

The emission spectra of O-dots suspended in aqueous media demonstrated large Stokes shifts (about 120 nm), which were much more pronounced than those of traditional fluorescent dyes. For example, the Stokes shift of fluorescein is about 30 nm. Measured values of Stokes shift were strongly dependent on the solvent used (see Supporting Information File 1, Figure S17). Upon replacement of the protic solvent (water) with an aprotic one (DMSO), the emission band excited at 350 nm shifts by 33 nm to longer wavelengths, with the intensity remaining virtually unchanged. The luminescence band excited at 405 nm shifts by 25 nm to shorter wavelengths, while the emission intensity increases more than tenfold. This excitation band-specific solvatochromic effect might have been due to the passivation of the particle surface by DMSO molecules [48].

### Biological properties of O-dots

#### Cellular toxicity

**Figure 4:**
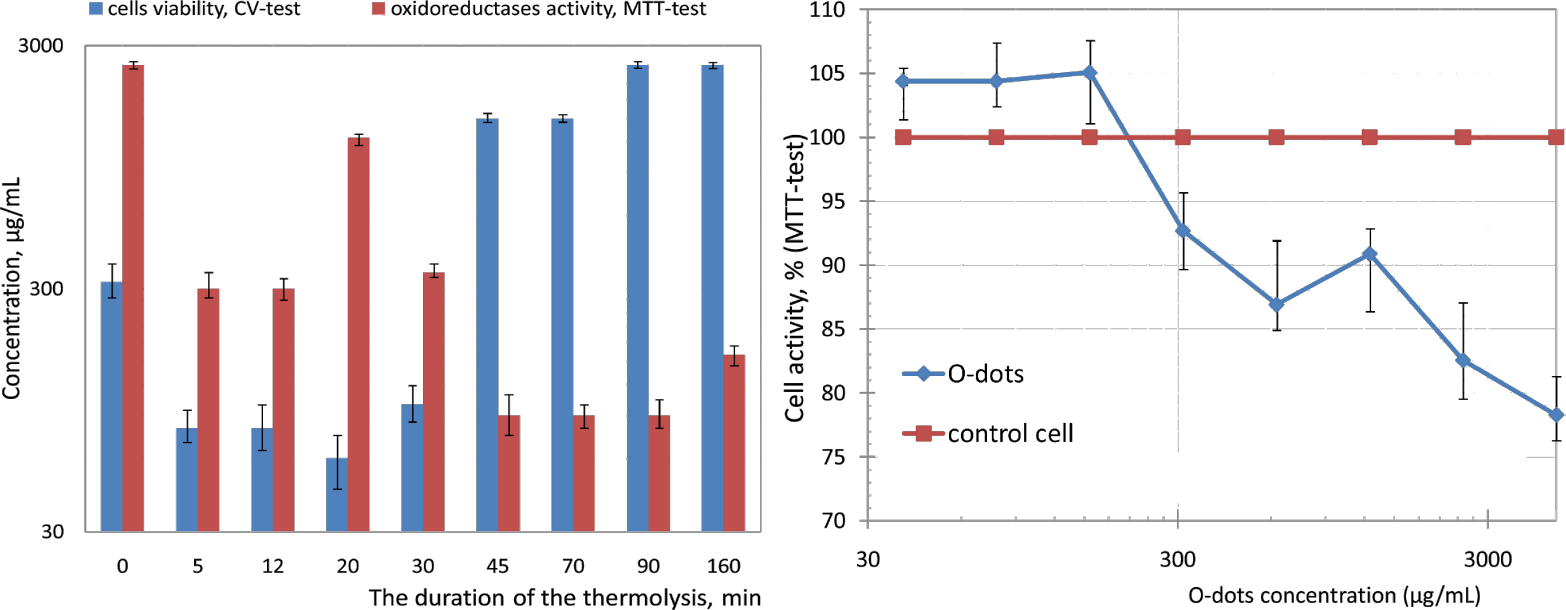
Left: Changes in the maximum tolerated concentration (MTC) and maximum concentration of metabolic activation (Max_met_) of a mixture of citric acid and urea (1:5 mol) heat-treated at 160 °C for 0–160 min for ST-cells. Right: Influence of different concentrations of O-dots, synthesized by heat treatment (160 °C, 160 min) of the mixture of citric acid and urea (1:5 mol), on the activity of NADP-H-dependent oxidoreductase enzymes in ST cells. The cells were exposed to O-dots for 24 h.

The results of studying the effect of O-dots samples on cell viability and metabolic activity of NADP-H-dependent oxidoreductases are shown in Figure 4 (for more details, see Supporting Information File 1, Table S1 and Table S2). It is demonstrated that a mixture of citric acid and urea, without heating (“0 min” sample), possessed some cytotoxicity. At concentrations greater than ca. 300 μg/mL, this sample decreased the number of living ST-cells. The enzymatic activity of the cells was not changed notably and the maximum concentration of metabolic activation (Max_met_) for the “0 min” sample was 2500 μg/mL.

After 5 min of heating, the toxicity of the mixture increased rapidly: MTC for “5 min” sample was ca. 80 μg/mL, for “20 min” ca. 60 μg/mL. Upon further heating, (up to 90 min), the toxicity of the mixture reduced gradually, and then remained stable at 2,500 μg/mL. The metabolic activity of cells was reduced in this case: Only at O-dot concentrations of about 100 μg/mL was the activity of NADPH-dependent mitochondrial oxidoreductase comparable to the activity of control cells; this concentration can be considered as Max_met_.

The observed effect is consistent with the proposed mechanism of O-dot formation upon heating a mixture of precursors. The "primary fluorophores", as low molecular weight aromatic compounds, have increased toxicity. Heat treatment leads to formation of polymeric structures, which are generally less toxic than the corresponding monomers.

Interestingly, the enzymatic activity of the cells incubated with different concentrations of O-dots, synthesized by heat treatment at 160 °C for 45, 70, 90 and 160 min, was maintained at a stable, low level: Max_met_ for "45 min", "70 min" and "90 min" samples was ca. 90 μg/mL, for the "160 min" sample about 160 μg/mL. Therefore, the synthesized O-dots do not cause destruction of the cell monolayer, but substantially inhibit the activity of the NADP-H-dependent oxidoreductase enzyme in ST cells, reducing their metabolism. The facts revealed may indicate the influence of O-dots on intracellular metabolism, which opens up the possibility of a regulation of intracellular redox processes. Synthesized O-dots could be effective as anti-tumour agents that actively suppress the metabolism of malignant cells.

Thus, at concentrations of up to 3,000 μg/mL, O-dots are non-toxic for normal cellular cultures, but inhibit the activity of NADP-H-dependent oxidoreductase enzymes of cells. Reducing the concentration of O-dots down to 700–900 μg/mL causes an inhibition of cell metabolism of less than 15%, relative to the control. The use of colloidal O-dot solutions with concentrations of less than 100 μg/mL has no effect on the viability and metabolic activity of the cells. This value is similar to the toxic levels typical for carbon dots obtained by other authors. For example, carbon dots synthesized via nitric acid oxidation of carbon soot reduced the viability of HepG2 cells by 20%, at concentrations higher than 100 μg/mL [49]. The graphene quantum dots prepared with graphene oxide as starting material were markedly toxic for MCF-7 and MGC-803 (human gastric cancer) cells at concentrations higher than 100 μg/mL [50]. “Green” carbon dots from coriander leaves extract became toxic for normal lung cells (L-132) or cancer cell line (A549) at concentrations higher than 500 μg/mL [51], the cancer cells being somewhat more sensitive.

Microcapsules decorated by O-dots had a very low cytotoxicity (Supporting Information File 1, Figure S18 and Figure S19). Obviously, this was due to the low concentration of O-dots in microcapsular delivery systems, which was significantly lower than both MTC and Max_met_.

#### Cellular staining by O-dots

**Figure 5:**
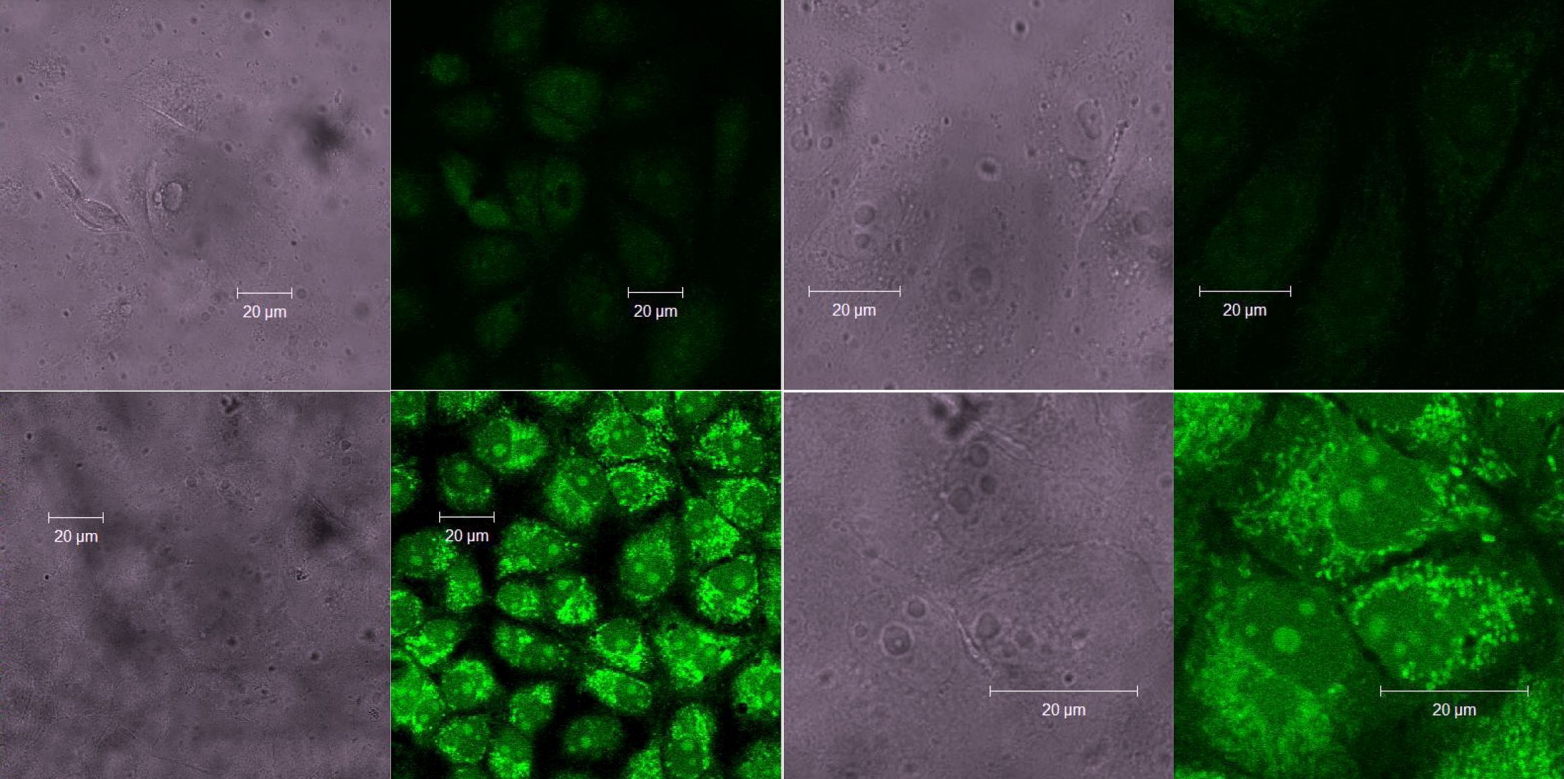
Fixed ST-cells stained with O-dots (50 μg/mL). Top: Normal cells. Bottom: Cells treated with hydrogen peroxide. Corresponding bright-field and confocal fluorescence (488 nm excitation) microscopy images with various magnifications are shown.

Results of cell staining are shown in Figure 5 and Figures S20–22 (Supporting Information File 1) for fixed and pristine (alive) cells, respectively. It is well known that cellular staining with fluorescent carbon dots is typically more effective for pre-fixed cells [52]. Data obtained indicate that the normal intact ST-cells were stained with O-dots (50 μg/mL) diffusely, and cellular luminescence was weak (Figure 5, top). Treatment with hydrogen peroxide (8 μg/mL, 15 min) initiated activation of oxidative stress in the cells; upon such treatment, the cells were stained more intensely (Figure 5, bottom). The use of O-dots allowed a clear visualization of the oxidative stress region: A bright glow was observed in the area of preferential localization of mitochondria in the perinuclear space. Increasing the number and size of nucleoli ("ribosomes factories”) correlates with the primary compensatory response of cells to oxidative stress during the first 15 min of contact with hydrogen peroxide, whose activation includes protein synthesis and ribosome formation. It should be noted that cells at the stage of division, or recently divided cells, absorb O-dots more actively, which may be due to the higher intensity of metabolic processes, and, as a consequence, they have a greater sensitivity to oxidative stress.

For comparison, a similar staining manipulation was carried out for the same ST cells treated with hydrogen peroxide, but without subsequent fixation (Supporting Information File 1, Figure S20). The micrographs obtained demonstrate that only some of the cells were stained, including those being in a state of apoptosis as a result of the treatment with hydrogen peroxide, or caught at the stage of division. O-dots were present in endosomes and formed apoptotic bodies. The use of a higher concentration of O-dots (125 μg/mL) enabled a more representative image of non-fixed ST-cells to be obtained (Supporting Information File 1, Figure S21). The findings suggest the possibility of the use of O-dots for the visual differentiation of normal cells and cells in a state of oxidative stress, as well as actively metabolizing cells.

Non-fixed malignant breast cancer cells (MCF-7S) absorb far more O-dots when used in the same concentration. A quite intense luminescence was observed for lamellipodia and ruffles of cancer cells, (Supporting Information File 1, Figure S22), and this can be attributed to facilitated penetration of the O-dots inside the tumour cells. This, in turn, can be attributed to violation of the regularity of the actin networks in the lamellipodia area, the appearance of a large number of “holes” in the sub-membrane layer of actin at the leading edge of the tumour cells and the destruction of the edge of the actin bundle, normally stabilizing the lateral cell edge [53]. Possibly, such differences in dyeing with O-dots of normal, stressed and malignant cells can be used for identification and characterization of tumour cells. In addition, the observed ability of O-dots to reduce the activity of NADP-H-dependent, intracellular oxidoreductases may find an application in the inhibition of the metabolic activity of the tumour cells. Synthesized O-dots combine the properties of dyes to provide differential staining of cells in various functional states, as well as the ability to inhibit enzymatic activity in highly metabolizing cells. This combination has a significant advantage within the framework of modern trends to create new combined anti-tumour agents [54].

Fixing cells generally locks cellular structures in place and makes it possible for larger molecules or nanoparticles to access the interior of the cell, for better staining: O-dots accumulate in the nucleoli of cells and perinuclear space, clearly separating the core-mantle boundary (Figure 5). Another advantage of the pre-fixation of cells is that, for the visualization of cell structures, a lower concentration of only about a third of O-dots is required. As in the case of intact, living cells, fixed cells are stained more strongly when subjected to oxidative stress.

#### LbL-microcapsules decorated by O-dots

**Figure 6:**
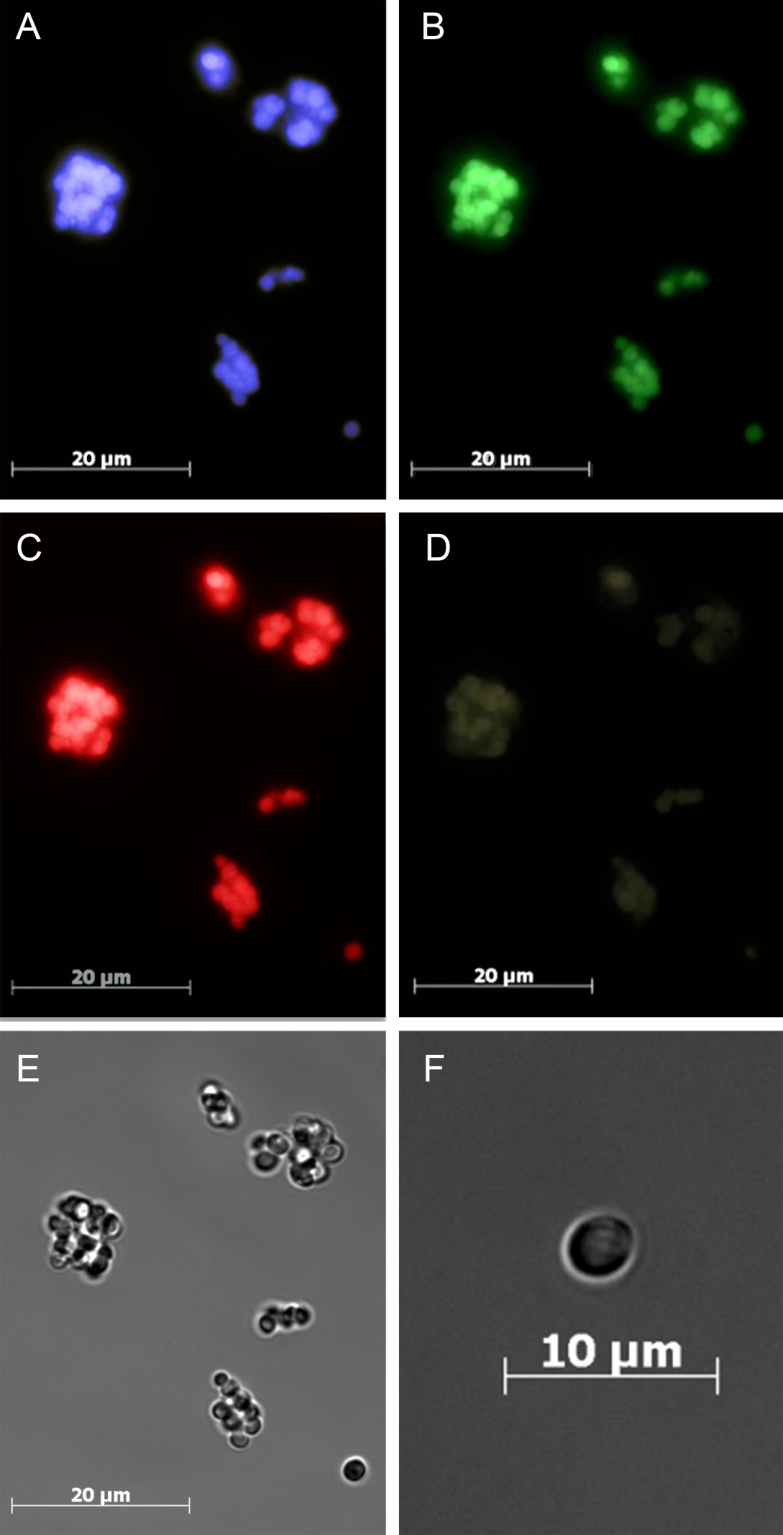
LbL-microcapsules decorated with O-dots. (A–D) Luminescent microscopy images: A – ex. 365 nm/em. 445 nm; B – ex. 490 nm/em. 525 nm; C – ex. 546 nm/em. 575 nm; D – ex. 578 nm/em. 603 nm; (E,F) phase contrast images.

Functionalization of microcapsules by O-dots leads to their bright luminescence (Figure 6). The apparent advantage of O-dots for capsule labelling is that O-dots (unlike traditional dyes) can be detected by using excitation light sources of different wavelengths. Thus, the standard optical setting for a luminescent microscope used in Rhodamine dye cell staining and visualization (ex. 546 nm/em. 575 nm) causes O-dots to emit in red (Figure 6A); the set used for DAPI staining (ex. 365 nm/em. 445 nm) causes O-dots to emit in blue (Figure 6B); the set used for FITC staining (ex. 490 nm/em. 525 nm) causes O-dots to emit in green (Figure 6C). With an appropriate combination of filters and mathematical data processing, such tunability makes it possible to pick out the unwanted background luminescence of biological objects, including the autofluorescence of cell components. Moreover, the multicoloured luminescence of the microcapsules decorated with O-dots can be detected in the cells in the presence of other individual luminescent dyes having a single excitation wavelength.

#### Monitoring of cellular uptake of LbL-microcapsules decorated with O-dots

**Figure 7:**
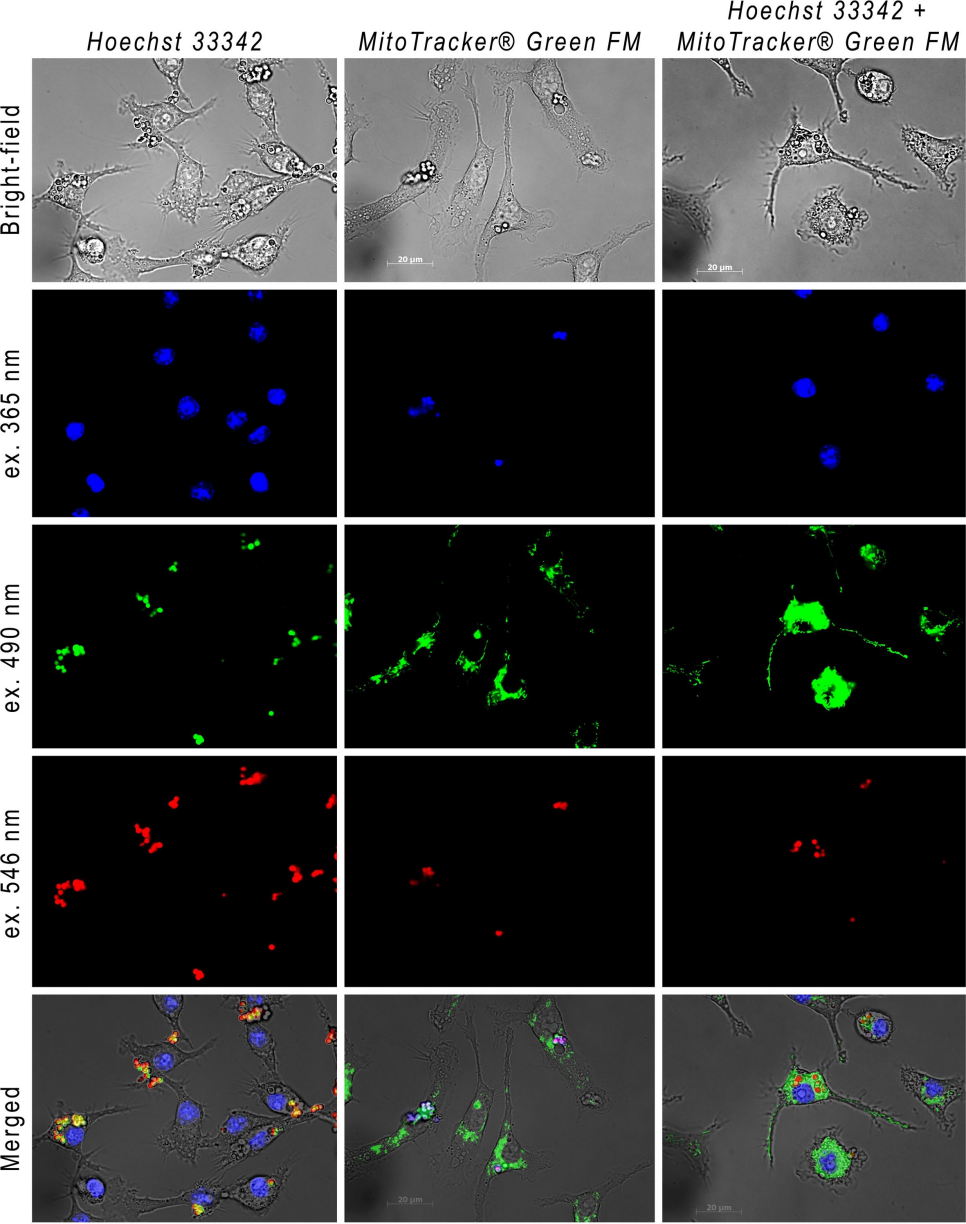
Murine macrophages upon uptake of O-dots decorated microcapsules, stained with Hoechst 33342, MitoTracker® Green FM or both dyes.

Murine macrophages readily take up microcapsules decorated with O-dots. By using different sets of filters, it is possible to register the multicoloured fluorescence of phagocytized microcapsules in vitro (Figure 7). Thus, this property allows staining of the cellular components with specific dyes simultaneously. For example, the luminescence of microcapsules can be registered in the presence of nuclear stain Hoechst 33342, mitochondrial stain MitoTracker^®^ Green FM, or both of them.

In conclusion, we have proposed a novel, one-pot, solvent-free method to synthesize luminescent organic dots (O-dots) with tunable luminescent properties. Synthesized O-dots combine the properties of dyes to provide differential staining of cells in various functional states, and the ability to inhibit enzymatic activity in highly metabolizing cells. The O-dots were successfully used to decorate multilayer polyelectrolyte microcapsules for cell staining.

## Experimental

### Measurements

Luminescent measurements were carried out on a Cary Eclipse (Varian) spectrofluorimeter equipped with a xenon lamp (150 W). Spectrophotometric measurements were made on a UV-2401PC (Shimadzu) spectrophotometer. Quantum yield (QY) was calculated according to IUPAC recommendation [55]:


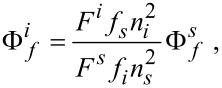


where 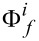 and 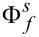 are the photoluminescence QY of the sample and that of the standard, respectively; *F^i^* and *F^s^* are the integrated intensities of sample and standard spectra, respectively (in units of photons); *f_x_* is the absorption factor (*f_x_* = 1 − 10^−^*^Ax^*, where “*A*” stands for absorbance); the refractive indices of the sample and reference solution are ni and ns, respectively; QY measurements were performed using fluorescein as standard.

### Synthesis of the O-dots

In all the experiments, the specified amount of citric acid (5 mmol) was placed in a Petri dish and dissolved in a tenfold (wt) amount of distilled water, and then the calculated amount of urea (0–25 mmol) was added. Some experiments (see Supporting Information File 1, Figure S12 and Figure S13) were performed with the addition of ammonia. After dissolving the components, the uncovered dish was placed into the ventilated oven, the excess of water was removed at 110 °C and then the temperature was increased to 160 °C (or to an otherwise specified temperature). The thermolysis was performed during the specified time interval (0–360 min, typically 120 min, see Figures 1–4 and Supporting Information File 1, Figures S5–S10, S12).

### Synthesis of the LbL-microcapsules decorated by O-dots

**Figure 8:**
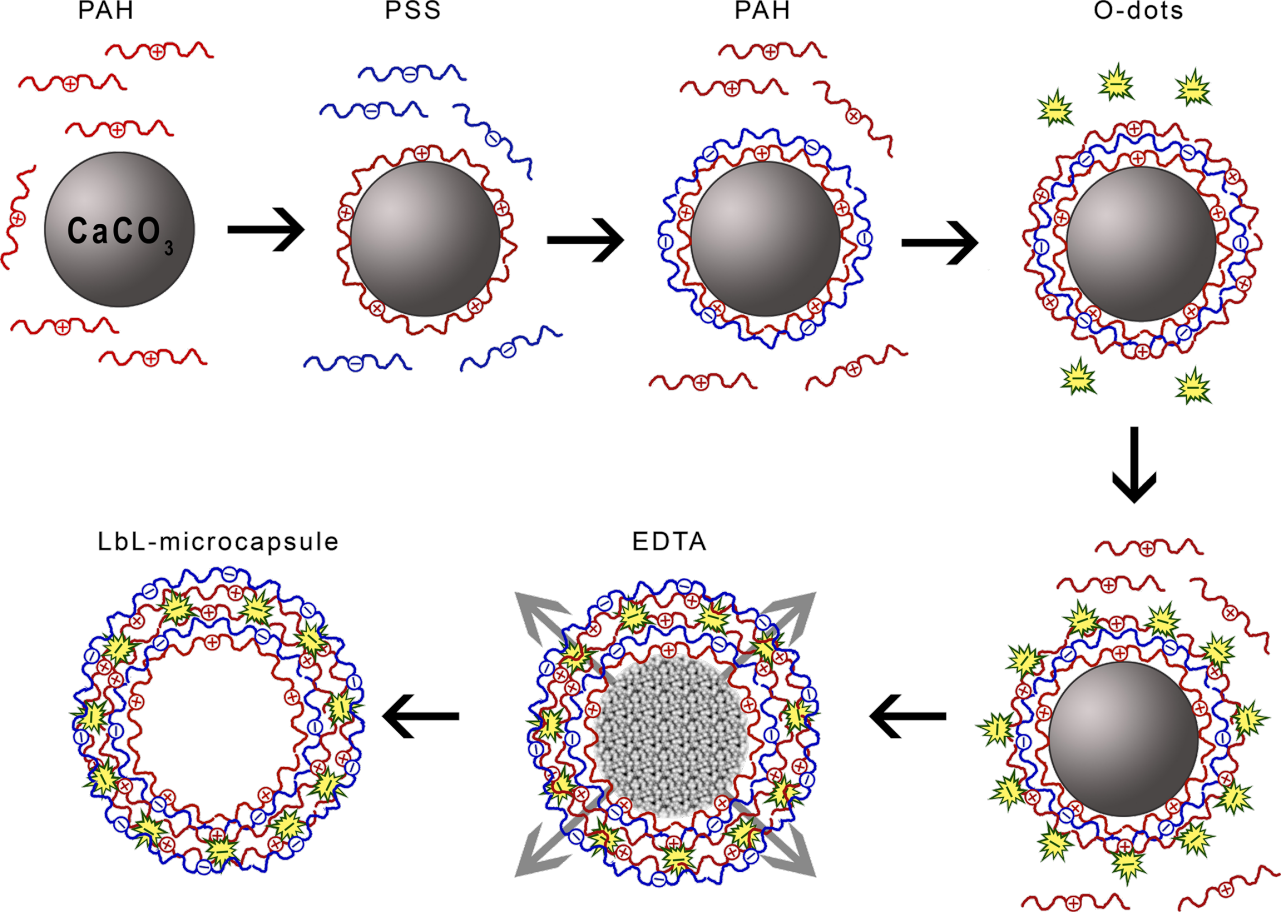
Schematic illustration of the formation of microcapsules: LbL-polyelectrolyte deposition, decoration with O-dots and subsequent CaCO_3_-core decomposition.

Polyelectrolyte microcapsules were fabricated by using a layer-by-layer (LbL) technique, followed by dissolution of the core material as described in [56–57], with some modifications: instead of one polyanionic layer on the surface of microcapsules, a layer of negatively charged O-dots was used (Figure 8).

Calcium carbonate microparticles were used as the template for fabrication of the nanocomposite shells. The first polyelectrolyte layer was made by adsorption of the positively charged poly allylamine hydrochloride (PAH) from 1 mg/mL solution in 0.15 M NaCl (15 min of incubation and shaking) on CaCO_3_ microparticles dispersed in this solution. The second layer was prepared by absorption of the negatively charged polystyrene sulfonate (PSS) from 1 mg/mL solution in 0.15 M NaCl (15 min of incubation and shaking). The third layer was made by adsorption of PAH again, and the O-dots were fixed on the positively charged polymeric surface. Then, the above described procedures were repeated. As a result, each particle was composed of a core and the following layers: PAH/PSS/PAH/O-dots/PAH/PSS (Figure 8).

The core–polyelectrolyte particles were washed three times with deionized water after each adsorption step. Finally, the calcium carbonate cores were dissolved in ethylenediaminetetraacetic acid (EDTA) for 30 min. The microcapsules were centrifuged and rinsed three times with EDTA, and then three times with pure water.

### Cell cultures

The effects of O-dots on living cells were studied using reference diploid epithelial swine testicular cell line (ST-cells), from the collection of the Institute of Veterinary Medicine, UAAS, and malignant breast cancer cells (MCF-7S), from the collection of the Kavetsky Institute of Experimental Pathology, Oncology and Radiobiology, NASU. We used a one-day culture of cells that were grown in a DMEM + RPMI medium (Sigma, USA) containing 7% FBS (fetal bovine serum; Sigma, USA), in the presence of kanamycin and gentamicin (Arterium, Ukraine), at a concentration of 40 μg/mL. Cultures formed a uniform monolayer of cells. Oxidative stress was induced by introducing into the cellular medium 3% hydrogen peroxide solution, at a final concentration of 8 μg/mL, in a well.

The cytotoxicity of microcapsules was studied using two cell lines. MNNG/HOS human osteosarcoma cells and RAW 264.7 murine macrophages were cultured as monolayers in a minimal essential medium supplemented with 10% fetal bovine serum and antibiotics (100 U/mL penicillin/streptomycin). All culture medium components were purchased from PanEco (PanEco, Russia). Cultures were incubated at 37 °C in air containing 5% CO_2_. Cells growing exponentially were harvested by a brief incubation with 0.25% trypsin–ethylenediaminetetraacetic acid (EDTA) solution (Gibco). The cellular uptake of microcapsules was studied using RAW 264.7 murine macrophage-like cell line.

### The cytotoxicity of the O-dots

The direct toxicity of O-dot samples synthesized by heat treatment at 160 °C for 0–160 min, and their precursors, was studied using reference diploid epithelial swine testicular cell line (ST-cells), by means of two tests [58], the crystal violet staining technique (CV assay) and the (3-(4,5-dimethylthiazolyl-2)-2,5-diphenyltetrazolium bromide) staining technique (MTT assay) [59]. The CV assay, with some modifications [60], was used to assess the total number of adhered cells. The MTT assay, with modifications [61], was used to assess the activity of NADP-H-dependent oxidoreductases which were present in the mitochondria and cytosol of the cells [61–62].

To determine the effect of heat treatment duration on the toxicity of O-dots, a set of samples consisting of 2 g of a mixture of citric acid and urea (molar ratio 1:5) were prepared by heating at 160 °C for 0 to 160 min. Then, each sample was dissolved in 40 mL of water and neutralized to pH 7.2 with ammonia. Ex tempore, doubly diluted in sterile distilled water, samples were introduced into the medium with the cells monolayer, at a volume ratio of 1:10, and incubated for 24 hours at 37 °C (Supporting Information File 1, Figure S23). After exposure, the cells were washed twice with Hanks' balanced salt solution (HBSS without phenol red, Gibco, Invitrogen), stained with crystal violet (λ = 540 nm) or MTT (λ = 492 nm), and the optical density in the wells was determined using a 96-well plate reader (Thermo/LabSystems Multiscan MS Microplate Reader, Finland).

The percentage of cells in experimental wells, relative to the intact control wells (which was taken as 100%), was calculated using the formula: (*D*_ex_/*D*_contr_) × 100, where *D*_ex_ is the optical density of the experimental wells, and *D*_contr_ is the optical density of the intact (control) wells. Statistical treatment of the data obtained was performed using BioStat 2009 Professional 5.8.1 software, in accordance with standard recommendations. Experimental data are presented as the median and interquartile range Me (LQ–UQ), where Me = median (50% percentiles), LQ = 25% percentiles and UQ = 75% percentiles. In the entire series, the number of experiments conducted was three.

The highest concentration that did not cause destruction of the cell monolayer (crystal violet stain) was adjudged to be the maximum tolerated concentration (MTC) of the sample. It is also known that the MTT assay is typically used for quantifying metabolically active cells, independently of proliferation [63]. Therefore, the highest concentration of sample that caused, in the treated cells, the activation of NADP-H-dependent oxidoreductases, not more than in the intact cells, was adjudged to be the metabolic maximum concentration (Max_met_). Each experiment was repeated three times, with four replications.

### The cytotoxicity of the microcapsules decorated with O-dots

The toxicity of the microcapsules was studied using two tests, the aforementioned MTT assay, and the lactate dehydrogenase (LDH) activity assay (Thermo Scientific™ Pierce™ LDH Cytotoxicity Assay Kit). For the LDH assay, cells were seeded in 96-well plates and cultured at 37 °C in an atmosphere containing 5% CO_2_. Six hours after cell seeding, the medium was replaced with the medium containing the microcapsules, with 1, 10, 50 or 100 capsules per cell. A positive control consisted of the cells without the addition of the microcapsules. A negative control consisted of the cells treated with Triton X-100 (10 µL). Then, 24 h after addition of the microcapsules, the level of lactate dehydrogenase in the culture medium was determined, according to the protocol of the manufacturer.

### Cellular staining

We used two methods for visualizing cells by synthesized O-dots: without fixing, and with fixing, using a mixture containing 2.5% glutaraldehyde (Sigma) and 4% paraformaldehyde (Sigma) in phosphate-buffered saline (pH 7.2, Sigma). The experiments were performed at room temperature.

Cells (5 × 10^4^) were grown on 15 mm glass coverslips. Staining of live unfixed cells that formed the monolayer on the surface of the coverslip was carried out ex tempore before rendering. O-dots were taken in 50 μg/mL or 125 μg/mL concentrations, (more than 50 times lower than the maximum tolerable concentration). Upon staining, the medium was removed and the cells were washed twice with Hanks' balanced salt solution (HBSS without phenol red, Gibco, Invitrogen). The coverslips with the cells were mounted in Attofluor cell chambers (Life Technologies). To model oxidative stress in the case of fixed cell staining, they were pretreated for 15 min with a solution of hydrogen peroxide, (the H_2_O_2_ concentration in the wells was 8 μg/mL). The cells were washed twice with PBS and fixed with the aforementioned fixative for 10 min. Afterwards, the cells were again washed twice with PBS, and incubated with О-dots at a concentration of 50 μg/mL. After 1 h, the medium was removed, and the cells were washed twice with PBS.

Microscopy studies were performed immediately after staining at room temperature. Fluorescent images were acquired using a LSM 510META (Carl Zeiss) confocal laser scanning microscope, equipped with a Plan-Apochromat 100×/1.4 Oil DIC objective. The excitation wavelength was 488 nm.

### Uptake of LbL-microcapsules

Cells were seeded in a 35 mm μ-Dish (Ibidi, Germany) and cultured in an atmosphere containing 5% CO_2_, at 37 °C. Six hours after cell seeding, the medium was replaced with the medium containing the microcapsules in the amount of 20 capsules per cell. After 24 h, cells were washed three times with PBS, and fluorescent microphotographs were made using Axiovert 200 (Zeiss, Germany). Cell nuclei were stained with Hoechst 33342, and mitochondria were stained with MitoTracker^®^ Green FM (Thermo Fisher Scientific).

### Statistical analysis

Experimental data are presented as the median and interquartile range Me (LQ–UQ), where Me = median (50% percentiles), LQ = 25% percentiles and UQ = 75% percentiles. In the entire series, the number of experiments conducted was five. Statistical differences within and between groups were verified using one-way analysis of variance (ANOVA).

## Supporting Information

File 1 Additional pictures and experimental data.
