# Supplementary material for: Facile fabrication of luminescent organic dots by thermolysis of citric acid in urea melt, and their use for cell staining and polyelectrolyte microcapsule labelling
Source: Beilstein J Nanotechnol. 2016 Dec 2;7:1905–17. doi: 10.3762/bjnano.7.182 (PMC5238650; doi:10.3762/bjnano.7.182)
Supplement: File 1 — Additional pictures and experimental data. [file Beilstein_J_Nanotechnol-07-1905-s001.pdf]

Supporting Information

for

Facile fabrication of luminescent organic dots by thermolysis of citric acid in urea melt, and their use for cell staining and polyelectrolyte microcapsule labelling

Nadezhda M. Zholobak[1], Anton L. Popov[2], Alexander B. Shcherbakov[1], Nelly R. Popova[2], Mykhailo M. Guzyk[3], Valeriy P. Antonovich[4], Alla V. Yegorova[4], Yuliya V. Scrypynets[4], Inna I. Leonenko[4], Alexander Ye. Baranchikov[5] and Vladimir K. Ivanov*[5,6]

Addresses: [1]Zabolotny Institute of Microbiology and Virology, National Academy of Sciences of Ukraine, Kyiv 03680, Ukraine; [2]Institute of Theoretical and Experimental Biophysics, Pushchino 142290, Russia; [3]Palladin Institute of Biochemistry NAS of Ukraine, Kyiv 01601, Ukraine; [4]Bogatsky Physico-Chemical Institute, National Academy of Sciences of Ukraine, Odessa 65080, Ukraine; [5]Kurnakov Institute of General and Inorganic Chemistry of the Russian Academy of Sciences, Moscow 119991, Russia and [6]National Research Tomsk State University, Tomsk 634050, Russia

Email: Vladimir K. Ivanov* - van@igic.ras.ru

* Corresponding author

Additional pictures and experimental data

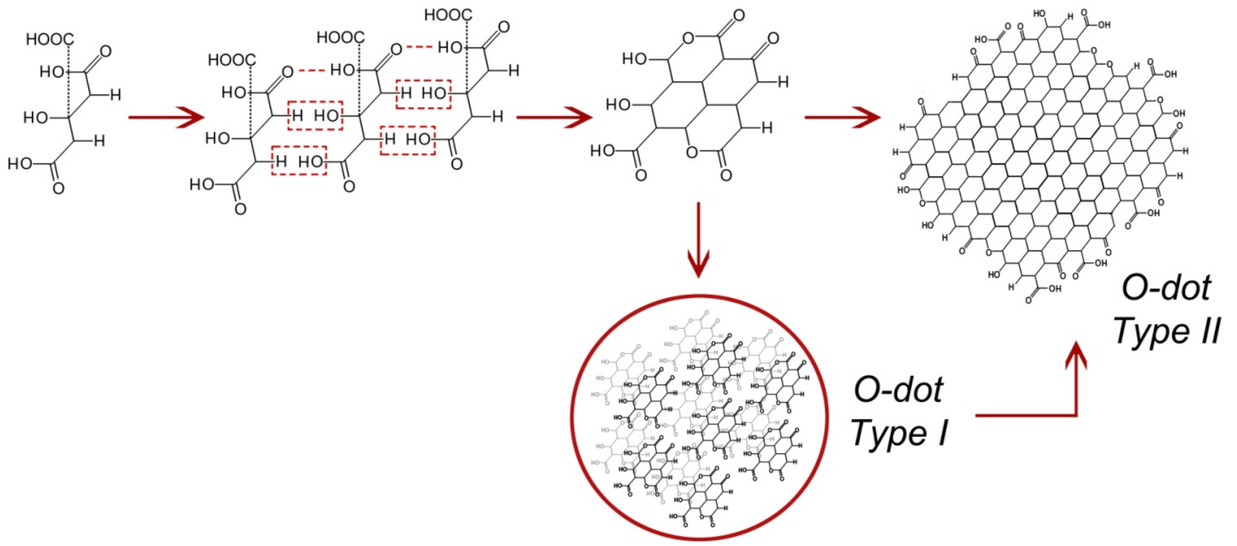

Figure S1: Formation mechanism of type-I and type-II carbon dots starting from citric acid. The intermediate “primary fluorophore” is a nominal (meaningless) unit.

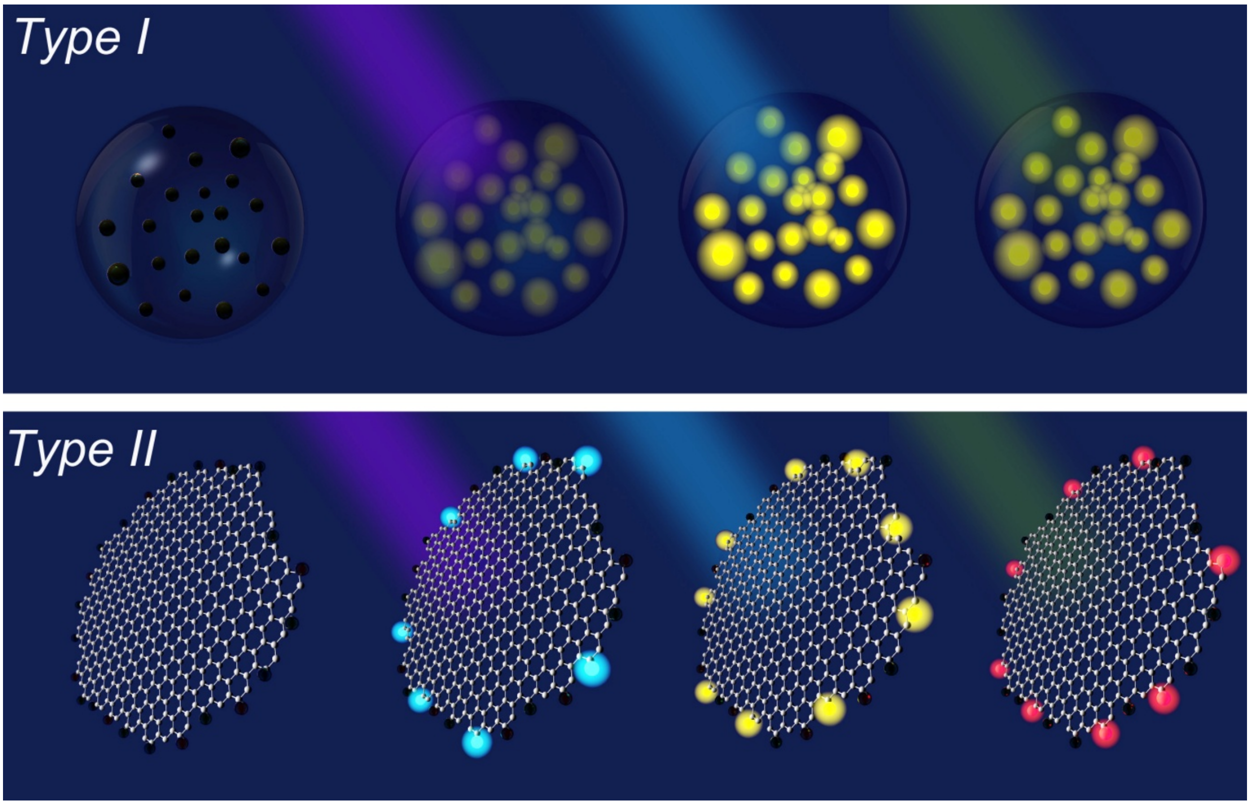

Figure S2: Schematic illustration of the O-dots’ luminescence upon excitation with light of different wavelengths.

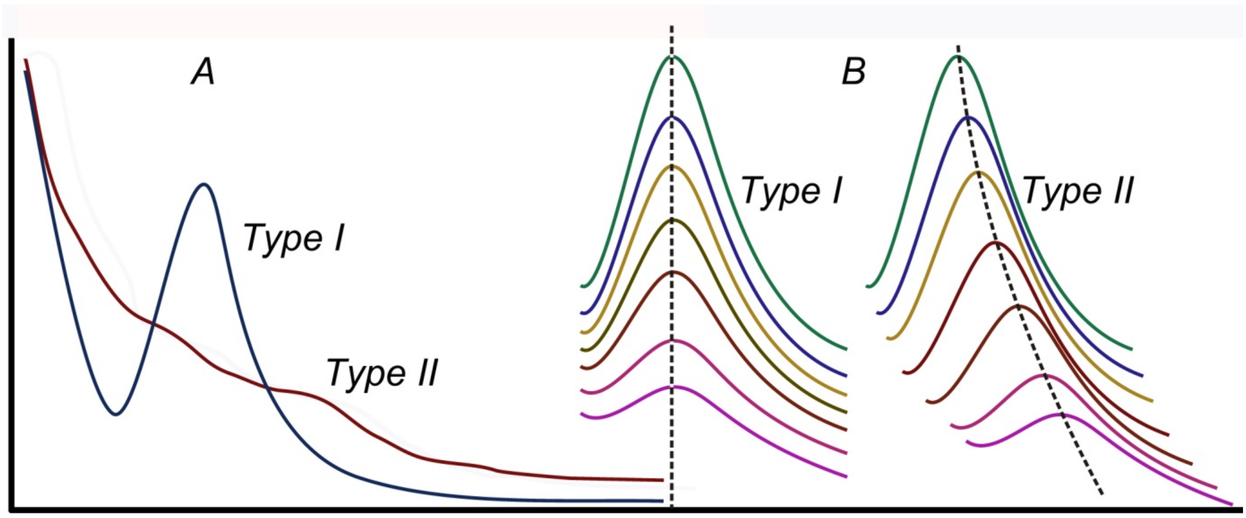

Figure S3: A – Typical absorption spectra of O-dots. B – Typical dependence of the luminescence spectra of O-dots on excitation wavelength.

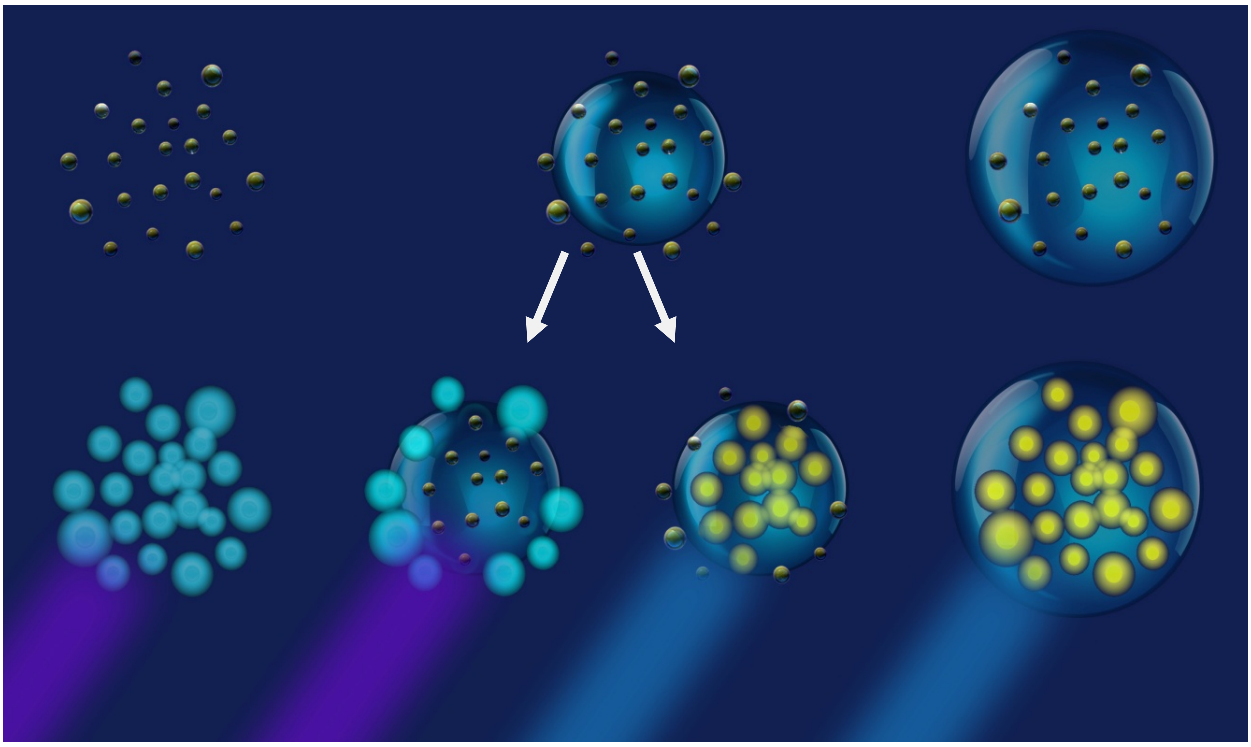

Figure S4: Schematic illustration of a possible change in the luminescence colour of the type-I O-dots during the process of their formation and growth; the luminescence of the intermediate cluster could be bimodal.

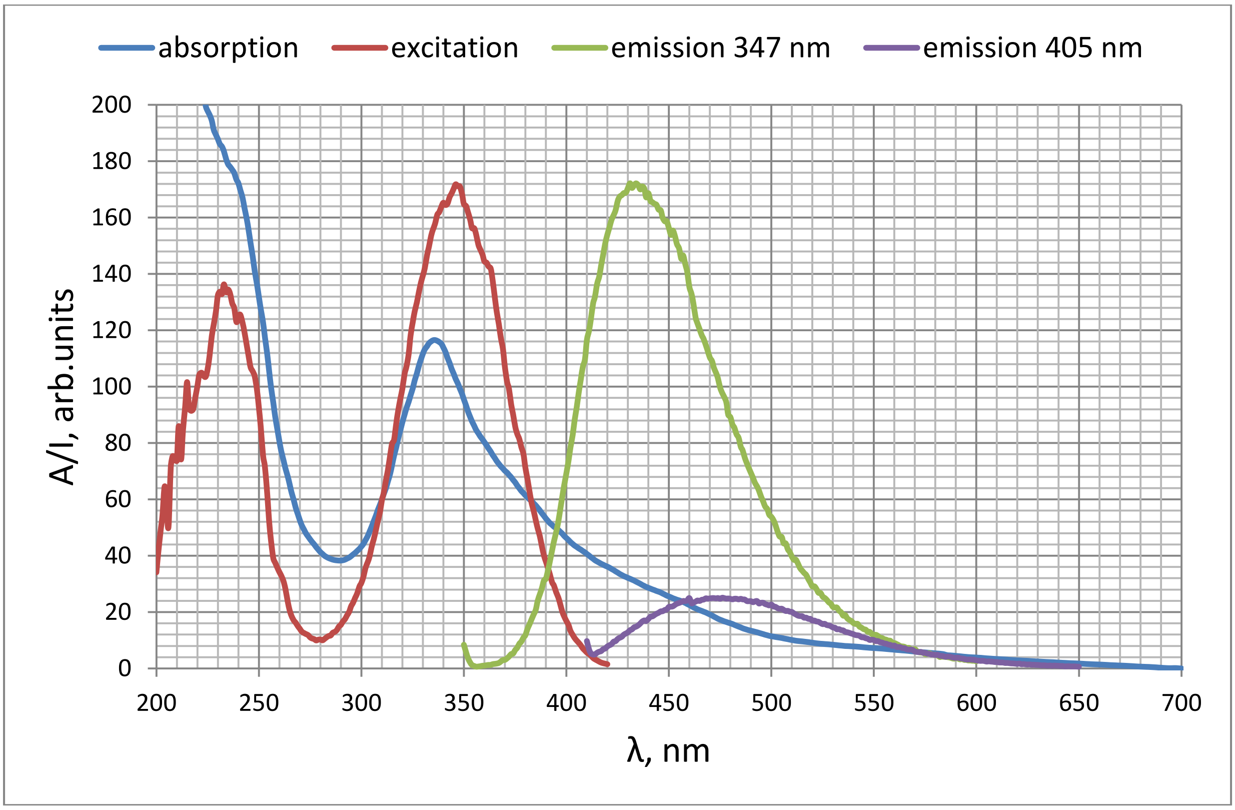

Figure S5: Absorption, excitation and emission spectra of the triammonium citrate heated at 160 °C for 120 min (aqueous solution, 2 µg/mL).

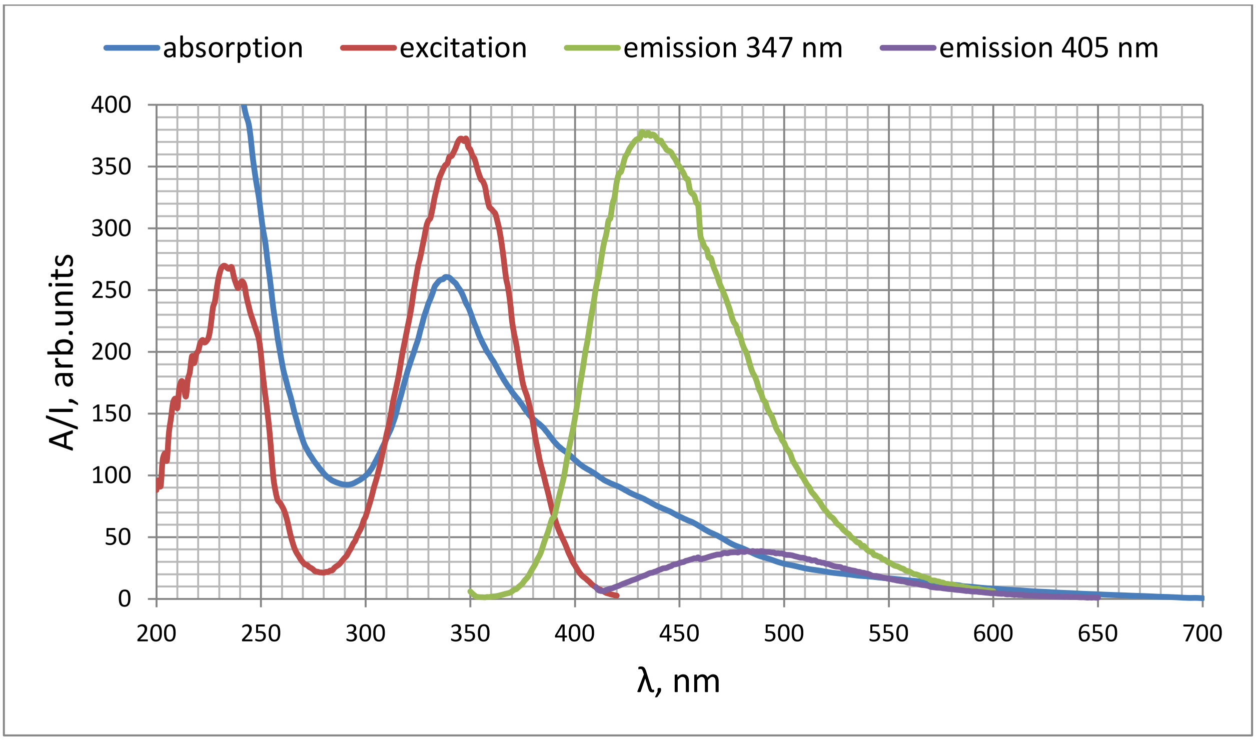

Figure S6: Absorption, excitation and emission spectra of the mixture of urea and citric acid (molar ratio 1:1) heated at 160 °C for 120 min (aqueous solution, 2 µg/mL).

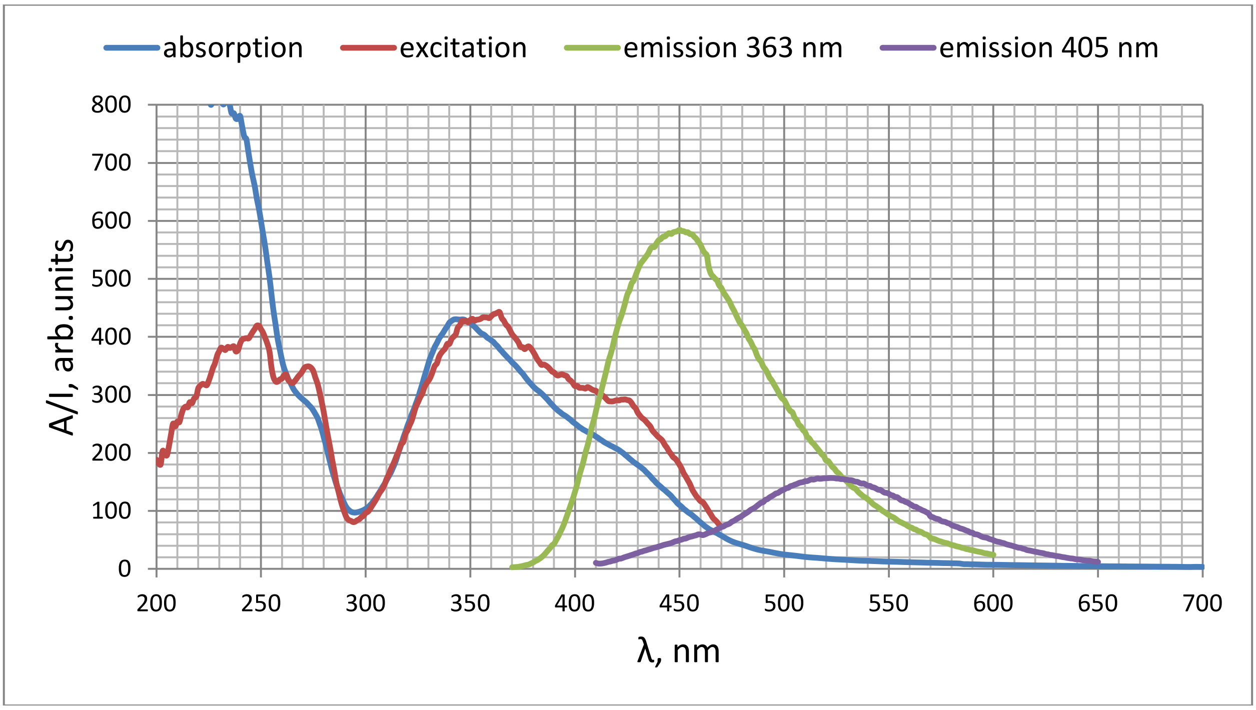

Figure S7: Absorption, excitation and emission spectra of the mixture of urea and citric acid (molar ratio 2:1) heated at 160 °C for 120 min (aqueous solution, 2 µg/mL).

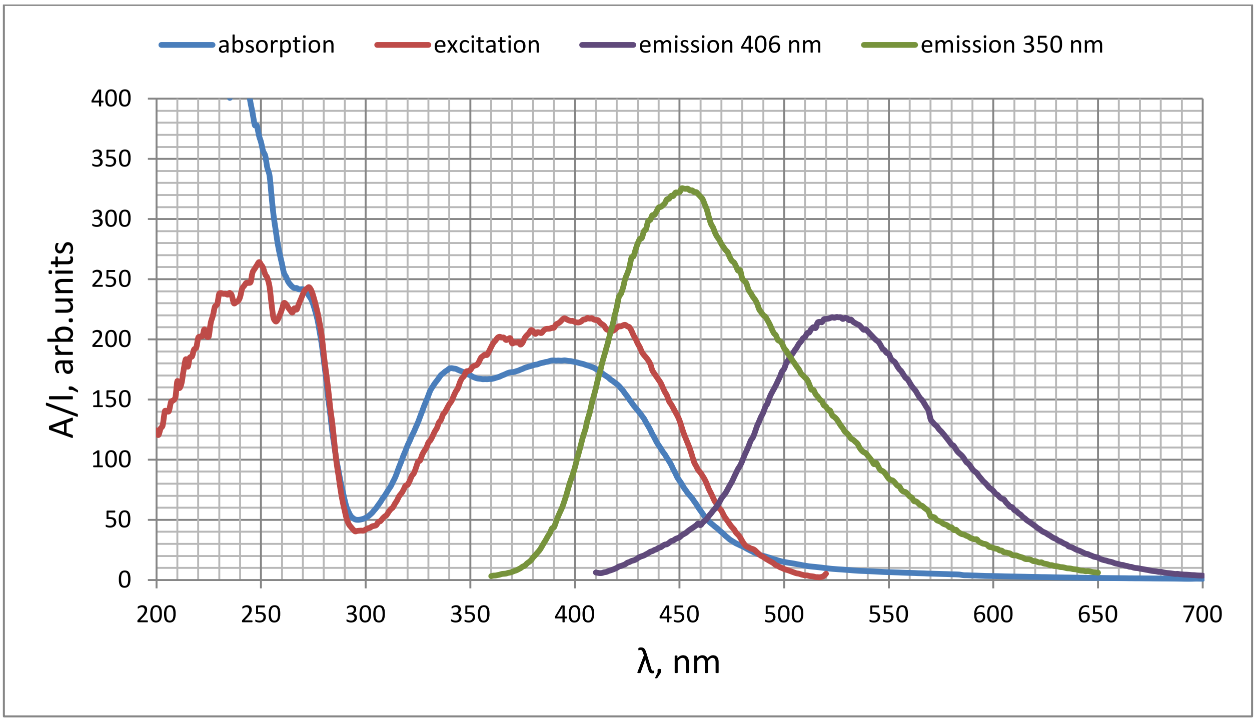

Figure S8: Absorption, excitation and emission spectra of the mixture of urea and citric acid (molar ratio 3:1) heated at 160 °C for 120 min (aqueous solution, 2 µg/mL).

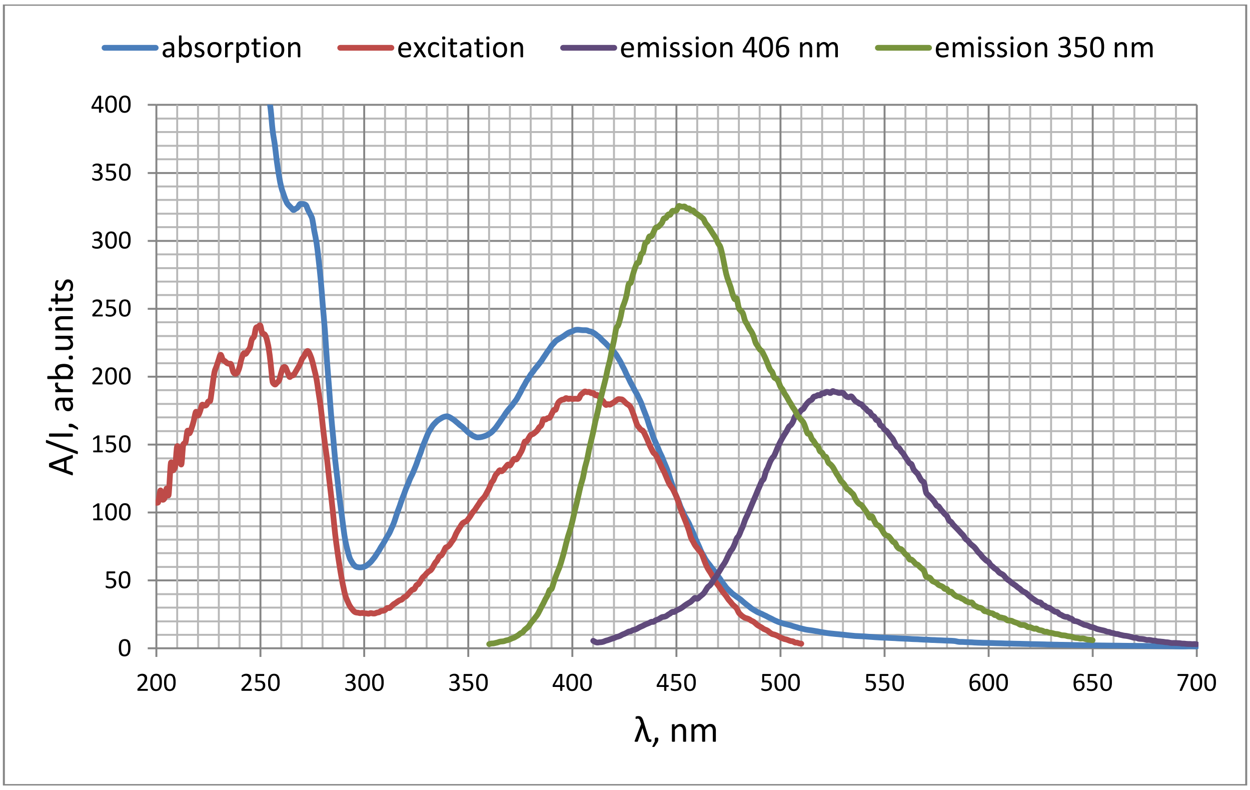

Figure S9: Absorption, excitation and emission spectra of the mixture of urea and citric acid (molar ratio 4:1) heated at 160 °C for 120 min (aqueous solution, 2 μg/mL).

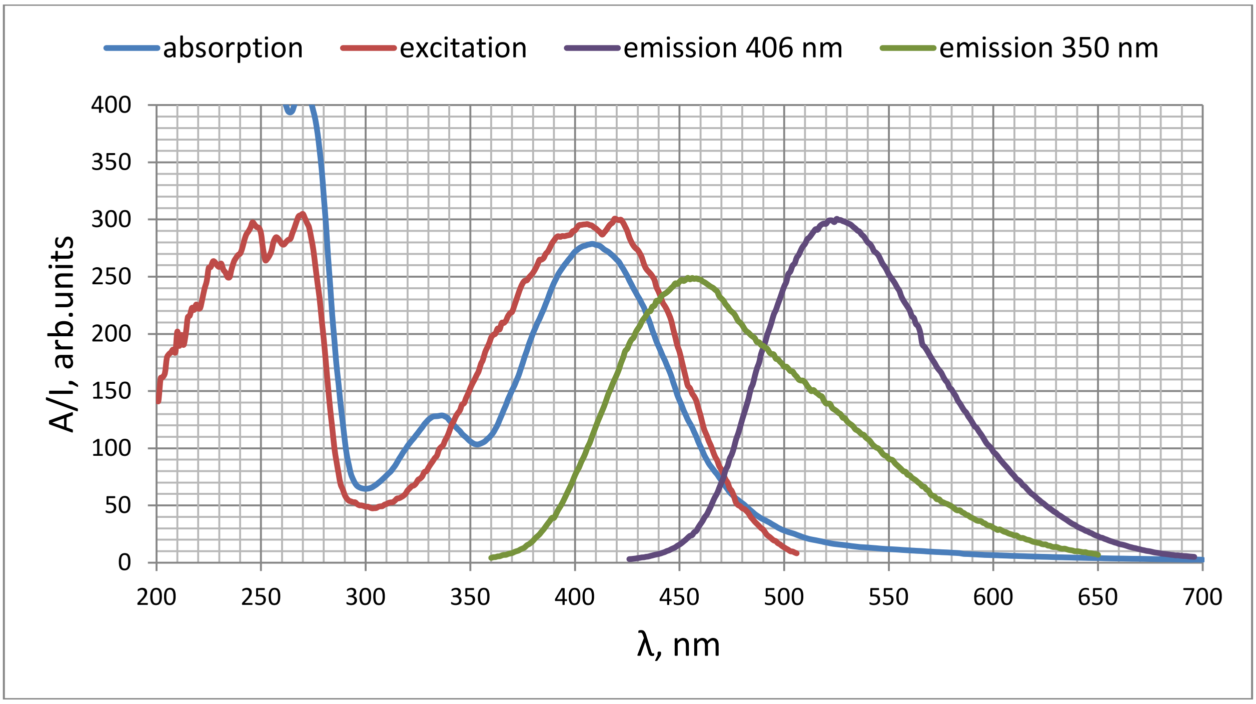

Figure S10: Absorption, excitation and emission spectra of the mixture of urea and citric acid (molar ratio 5:1) heated at 160 °C for 120 min (aqueous solution, 2 μg/mL).

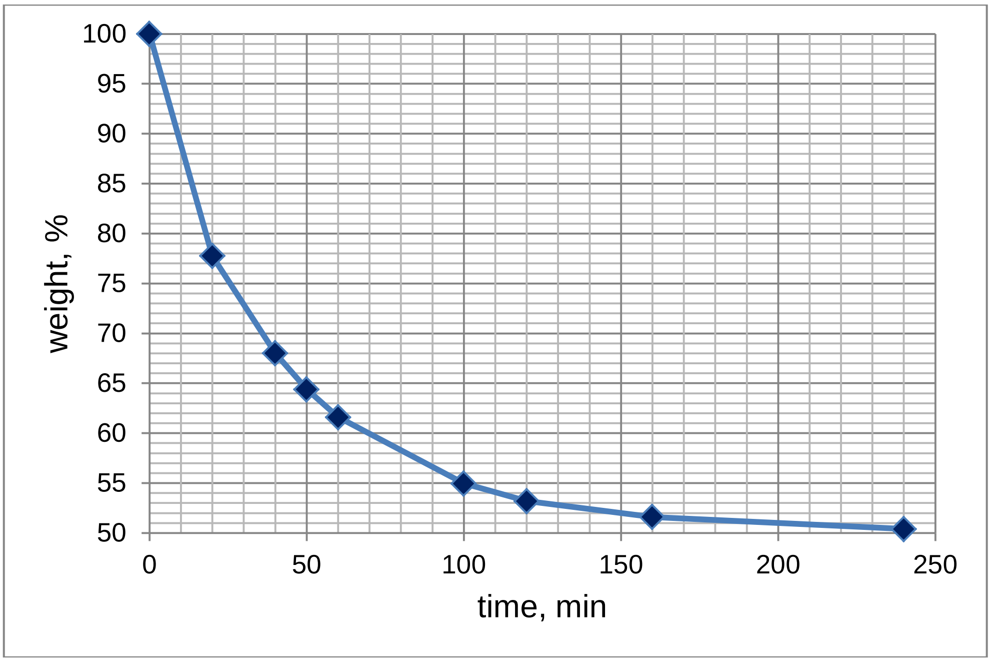

Figure S11: The dynamics of the weight loss of the citric acid and urea (1:5 mol) mixture during heating at 160 °C.

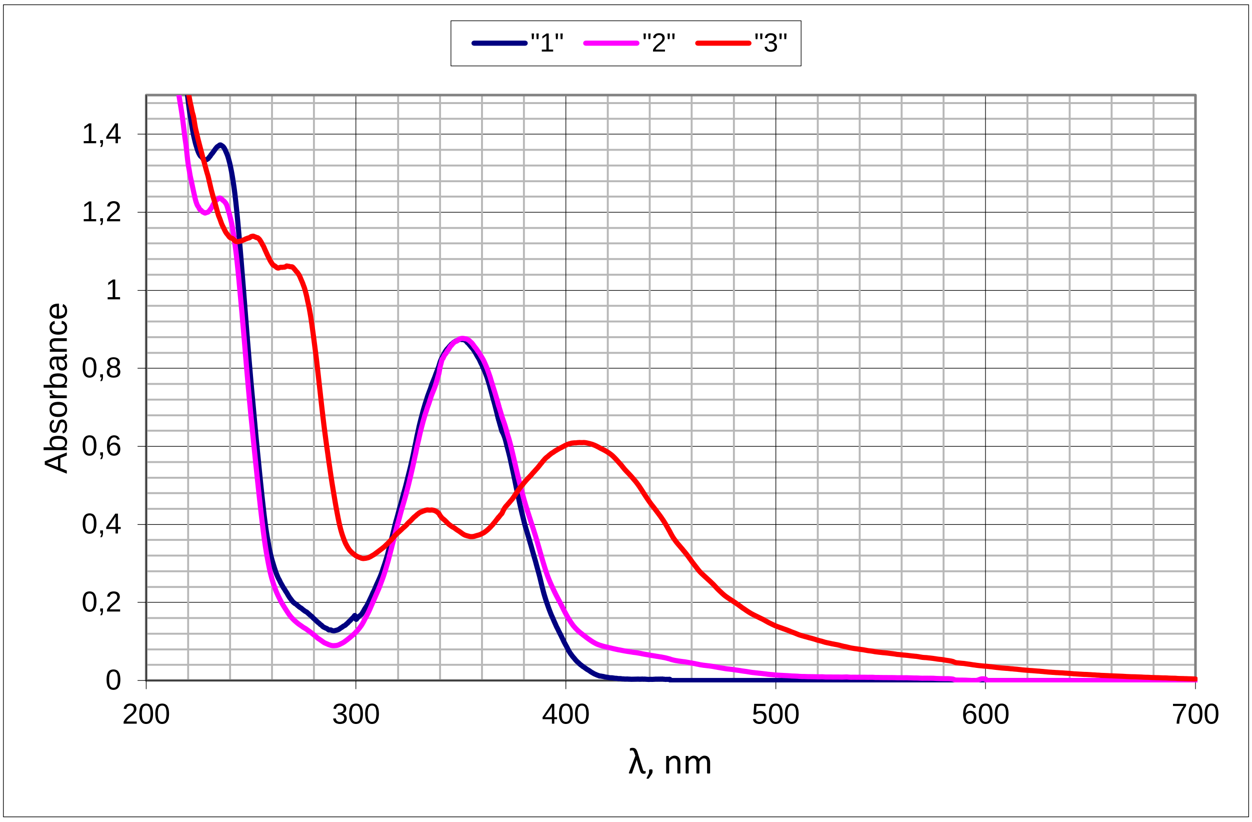

Figure S12: UV-absorption spectra of ammonium citrazinate (1), and the same compound heated at 160 °C for 120 min in the absence (2) and in the presence (3) of urea excess.

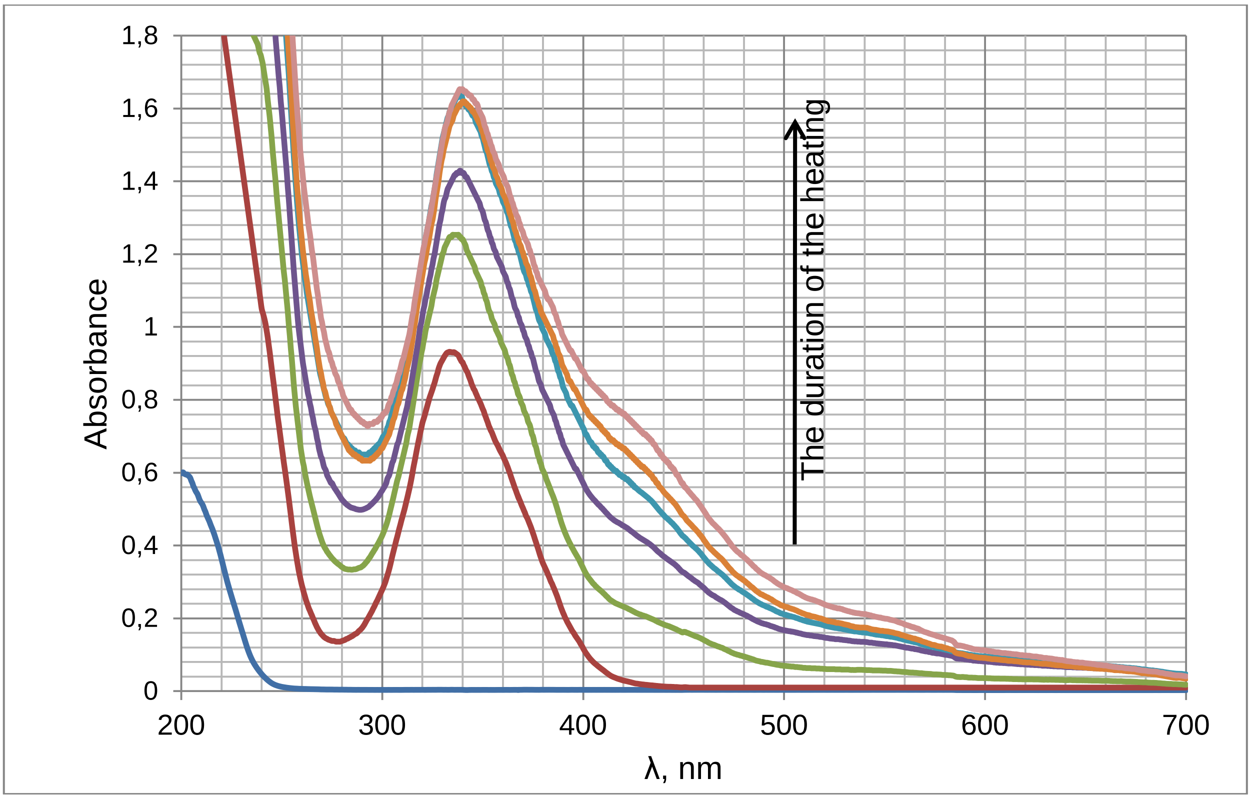

Figure S13: Changes in the absorption spectra of triammonium citrate upon heating at 160 °C for 0–360 min.

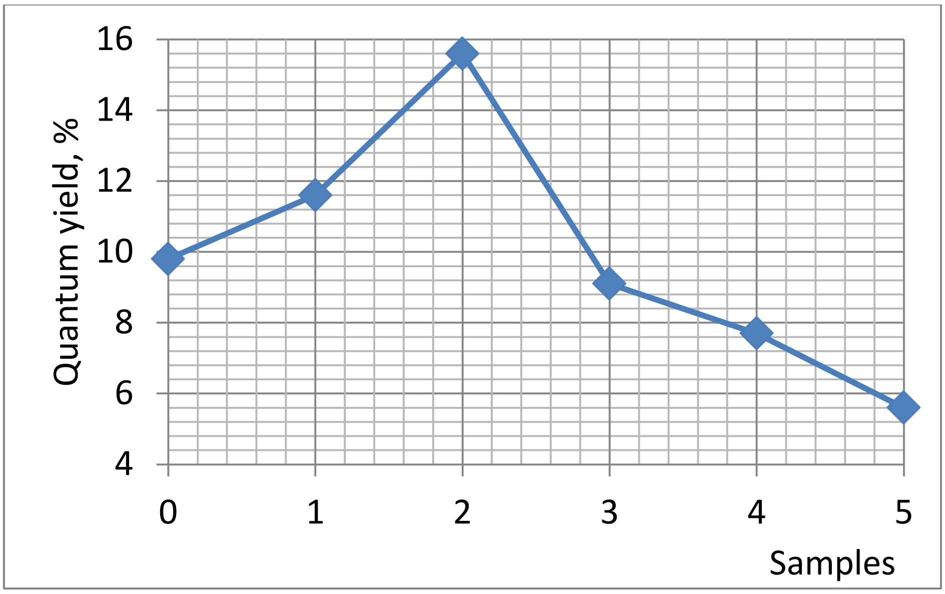

Figure S14: Quantum yields of the urea and citric acid mixtures heated at 160 °C for 120 min at optimal excitation wavelengths: "0", "1" – ex. 347 nm/em.431 nm; "2" – ex. 363 nm/em. 450 nm; "3", "4" – ex. 406 nm/em. 525 nm, "5" – ex. 406 nm/em. 529 nm. The number of samples corresponds to the urea:citric acid molar ratio; sample "0" is a product of thermal treatment of triammonium citrate under the same conditions.

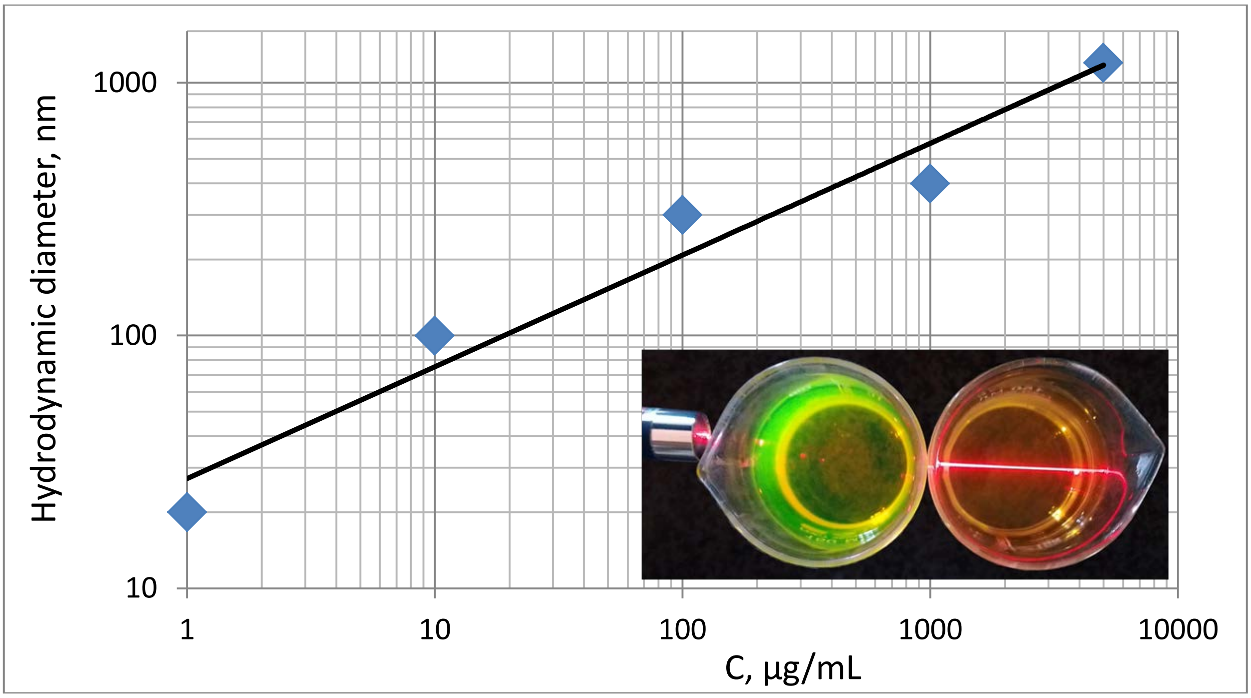

Figure S15: Dependence of O-dots' mean hydrodynamic diameter on their concentration in aqueous sol. Insert: Red laser beam in the 100 µg/mL fluorescein solution (left, no trace), and in the 100 mg/mL O-dots sol (right, Tyndall effect).

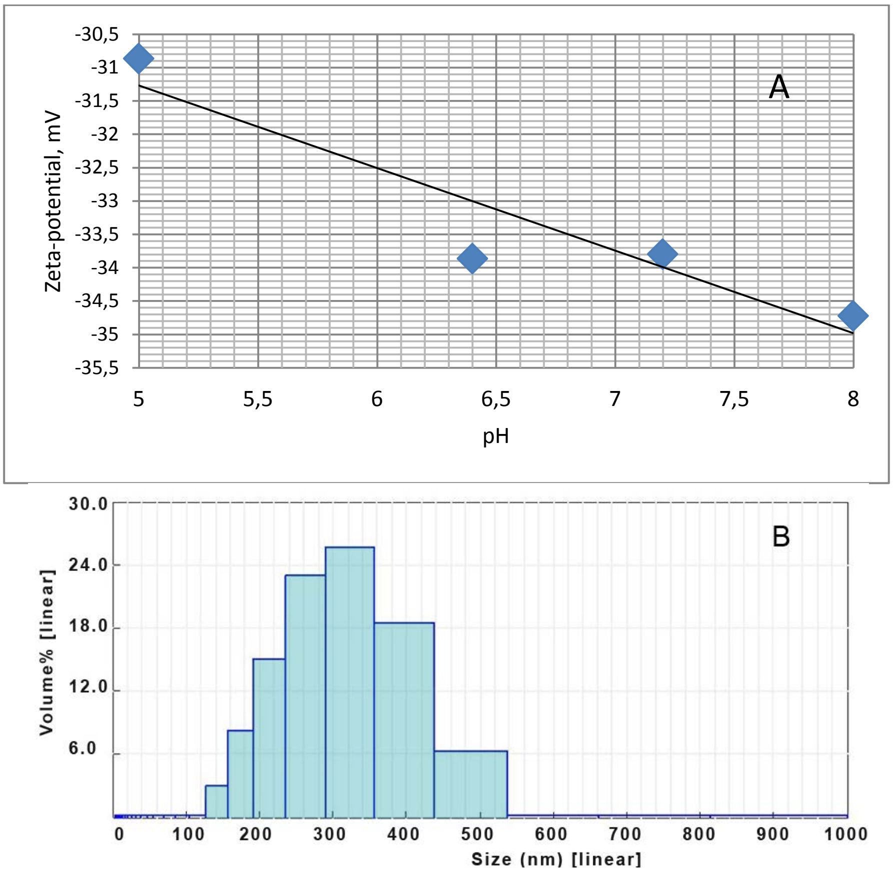

Figure S16: A – Dependence of O-dots' zeta-potential on the pH of aqueous sol; B – size distribution of hydrodynamic diameters of O-dots taken in 100 µg/mL concentration.

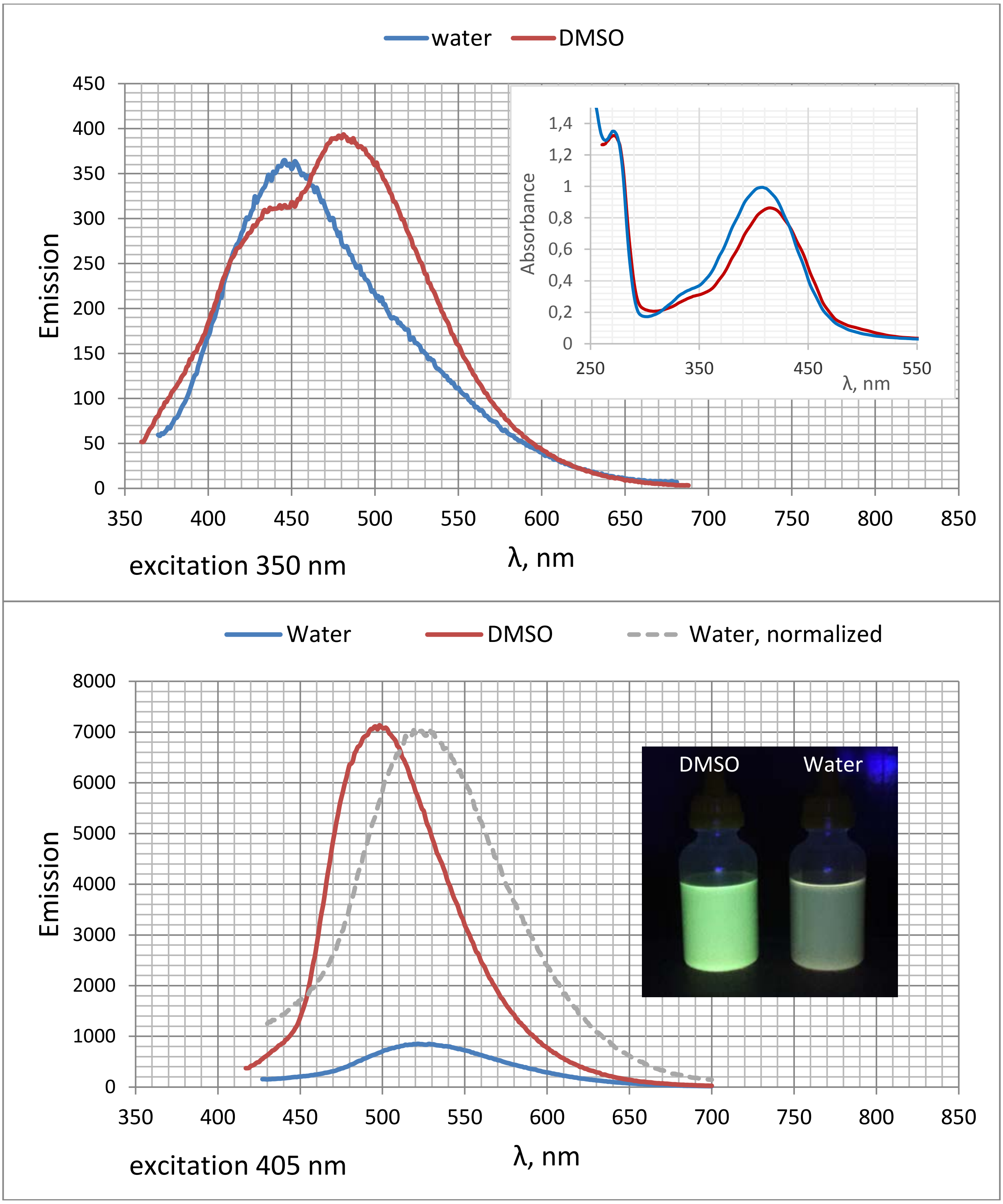

Figure S17: Luminescence spectra (350 nm and 405 nm excitation) of the O-dots in DMSO and water (relative and normalized). Inset: above – UV-absorption spectra of the O-dots in DMSO and water, below – appearance of equimolar sols of O-dots in DMSO and water under UV lamp.

Table S1: The number of adherent ST-cells upon 24 hours' exposure with different concentrations of O-dots synthesized from citric acid:urea (1:5 mol) mixtures, heated for different time intervals.

Time, min→ µg/mL↓	0	5	12	20	30	45	70	90	160	Cells control
5000	27.33	19.29	23.79	40.84	39.23	53.38	60.45	55.63	61.41	100.00
2500	57.23	93.25	87.14	91.00	85.21	95.50	91.96	103.22	130.23	100.00
1250	82.32	97.43	87.46	84.24	84.50	109.32	107.40	95.18	110.29	100.00
625	94.86	96.14	93.89	69.45	82.32	107.40	113.83	95.50	109.00	100.00
312	100.64	91.61	92.57	73.63	87.14	106.75	97.53	98.39	113.18	100.00
156	106.43	87.14	88.10	88.75	90.68	109.32	98.42	97.11	106.75	100.00
78	109.00	99.04	98.39	96.78	107.07	98.39	106.11	93.25	115.76	100.00
39	98.82	102.25	97.46	116.40	98.18	108.04	111.58	104.50	112.22	100.00

Table S2: The activity of NADP-H-dependent mitochondrial oxidoreductases in ST-cells upon 24 hours' exposure with different concentrations of O-dots synthesized from citric acid:urea (1:5 mol) mixtures, heated for different time intervals.

Time, min→ µg/mL↓	0	5	12	20	30	45	70	90	160	Cells control
5000	93.14	72.22	80.09	81.89	62.99	63.44	71.32	71.32	78.29	100.00
2500	101.91	93.14	93.14	93.81	75.82	76.72	75.59	72.89	82.56	100.00
1250	101.01	91.11	94.04	96.51	93.14	77.17	76.04	79.42	90.89	100.00
625	99.66	94.49	91.56	100.56	93.36	77.84	78.07	78.07	83.91	100.00
312	105.29	99.89	104.61	99.89	104.16	86.39	83.46	87.06	92.69	100.00
156	103.94	100.34	97.64	97.86	103.26	86.61	84.59	88.86	105.06	100.00
78	100.56	99.44	102.36	103.94	98.76	105.06	105.74	101.46	104.39	100.00
39	99.66	99.21	96.06	97.64	96.96	102.81	101.01	101.91	104.39	100.00

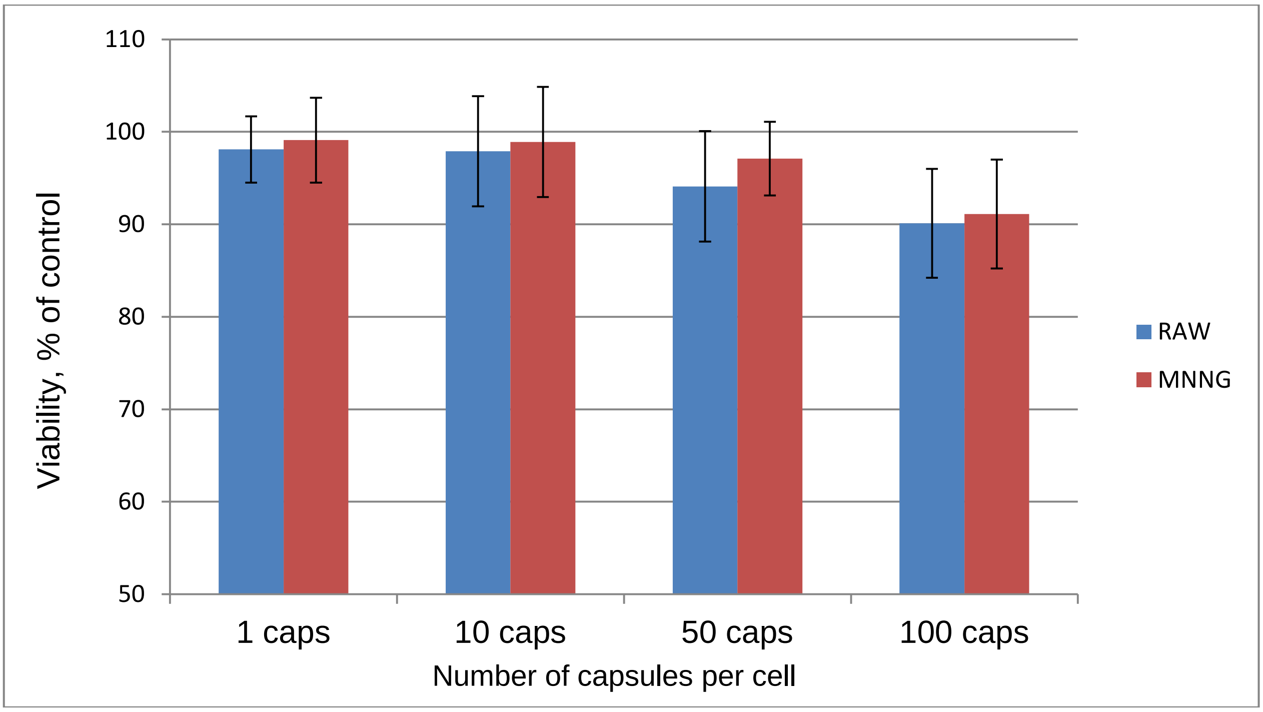

Figure S18: MTT assay upon 24 hours' incubation of the cells with microcapsules decorated with O-dots.

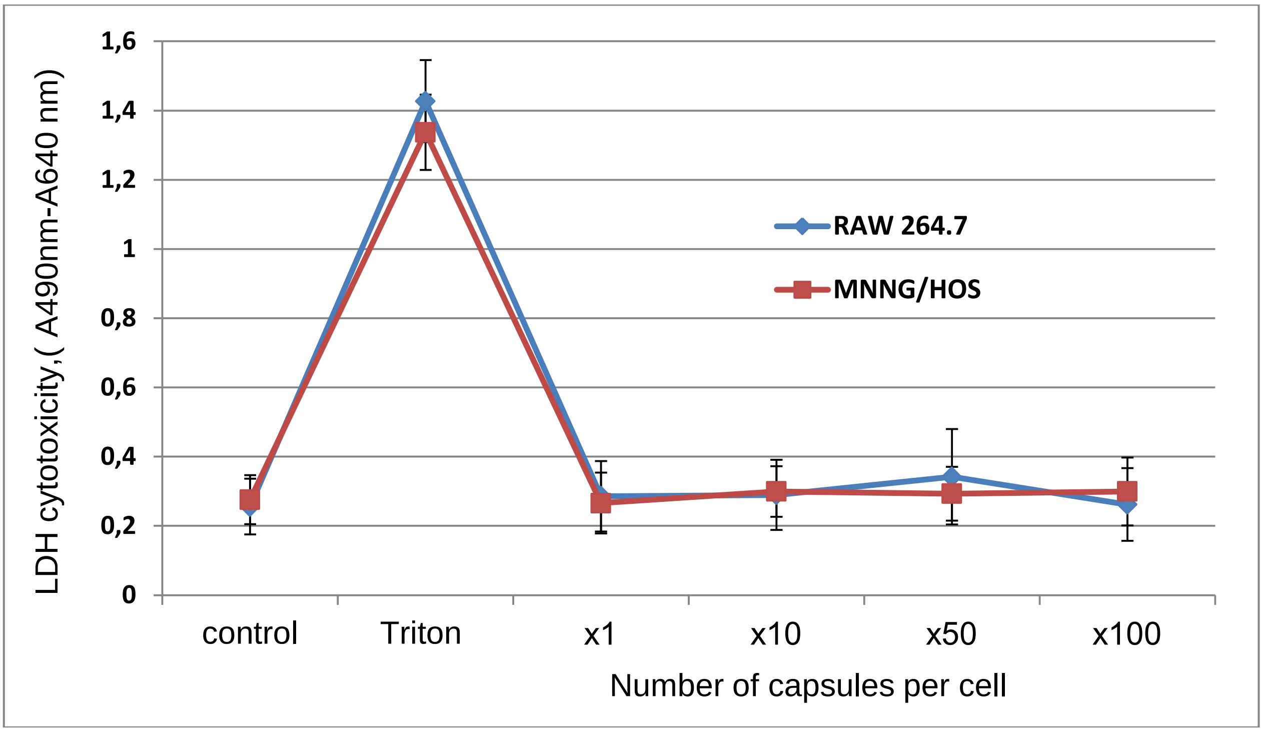

Figure S19: LDH assay upon 24 hours' incubation of cells with microcapsules decorated with O-dots.

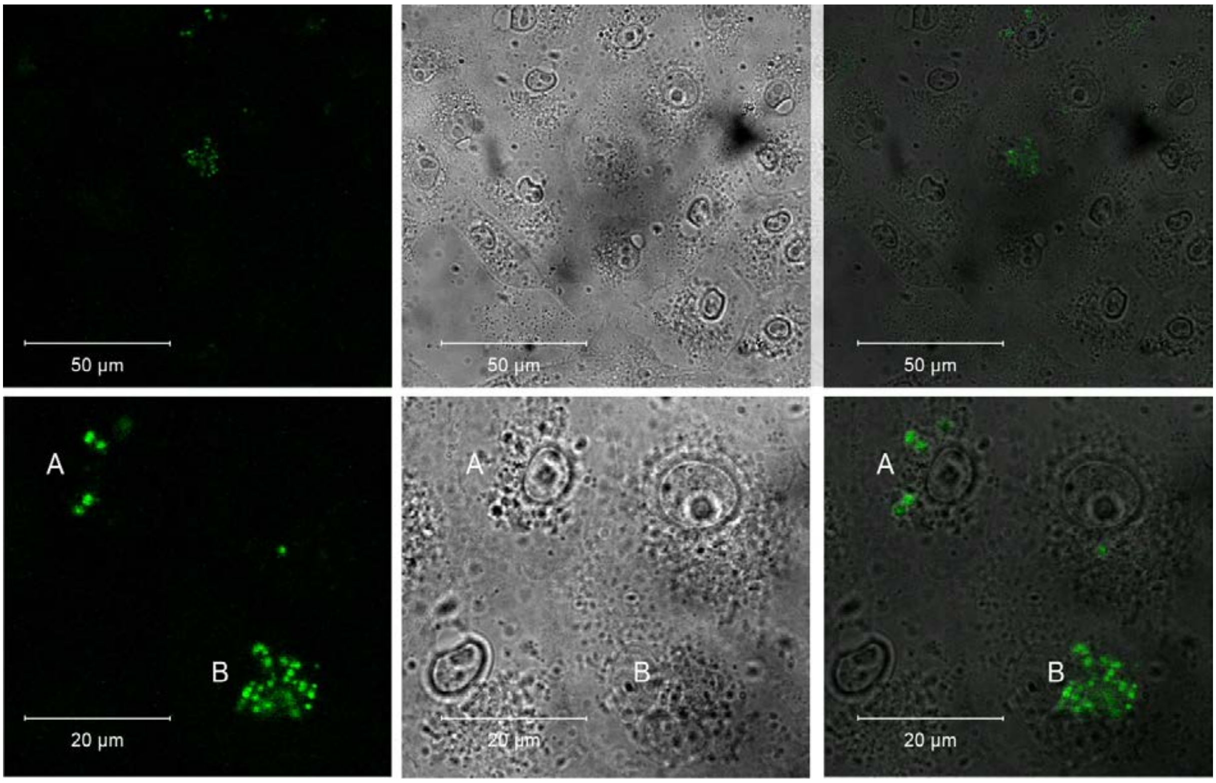

Figure S20: ST cells treated with hydrogen peroxide and stained by O-dots (50 µg/mL) without fixation. Left to right: fluorescence microscopy image (488 nm excitation), bright-field microscopy image, overlay image. The first row – standard image of the cells; the second row – enlarged image. A - Stained apoptotic bodies; B - Cell destroyed in the dividing stage.

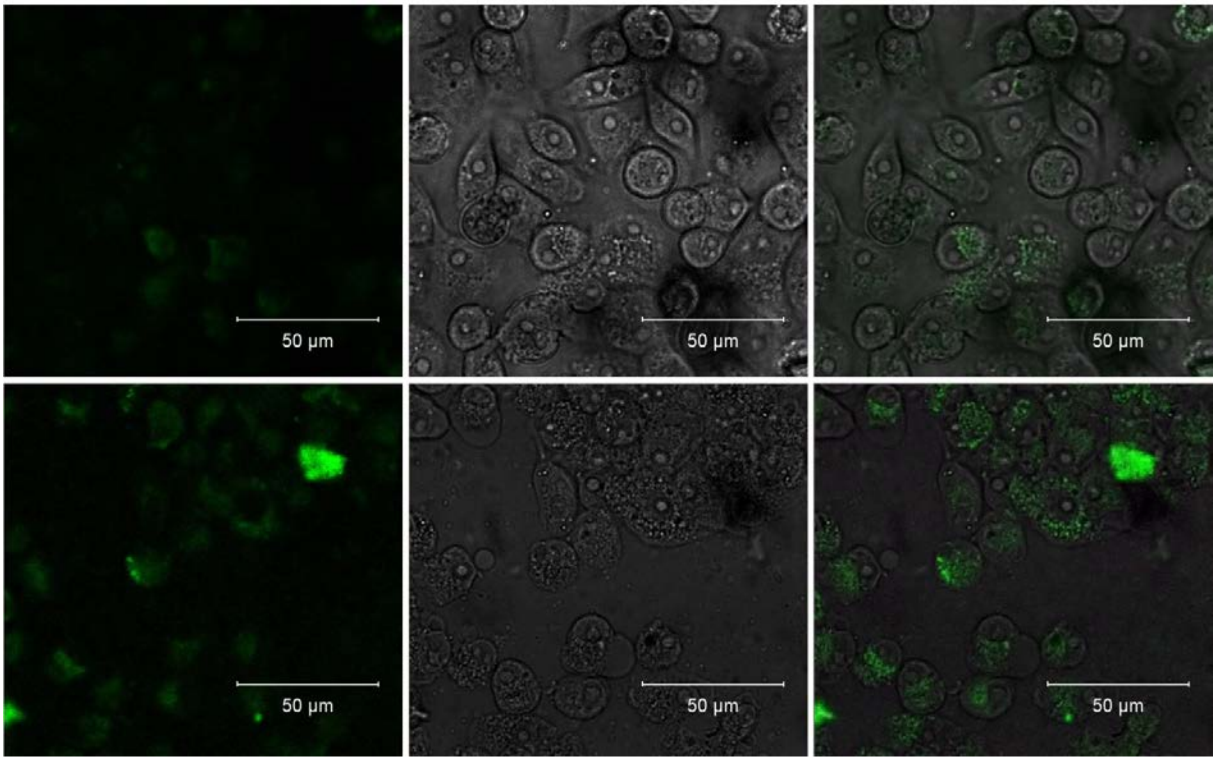

Figure S21: ST cells stained by O-dots (125 µg/mL) without fixation. Left to right: fluorescence microscopy image (488 nm excitation), bright-field microscopy image, overlay image. The first row – intact healthy cells; the second row – cells treated with hydrogen peroxide.

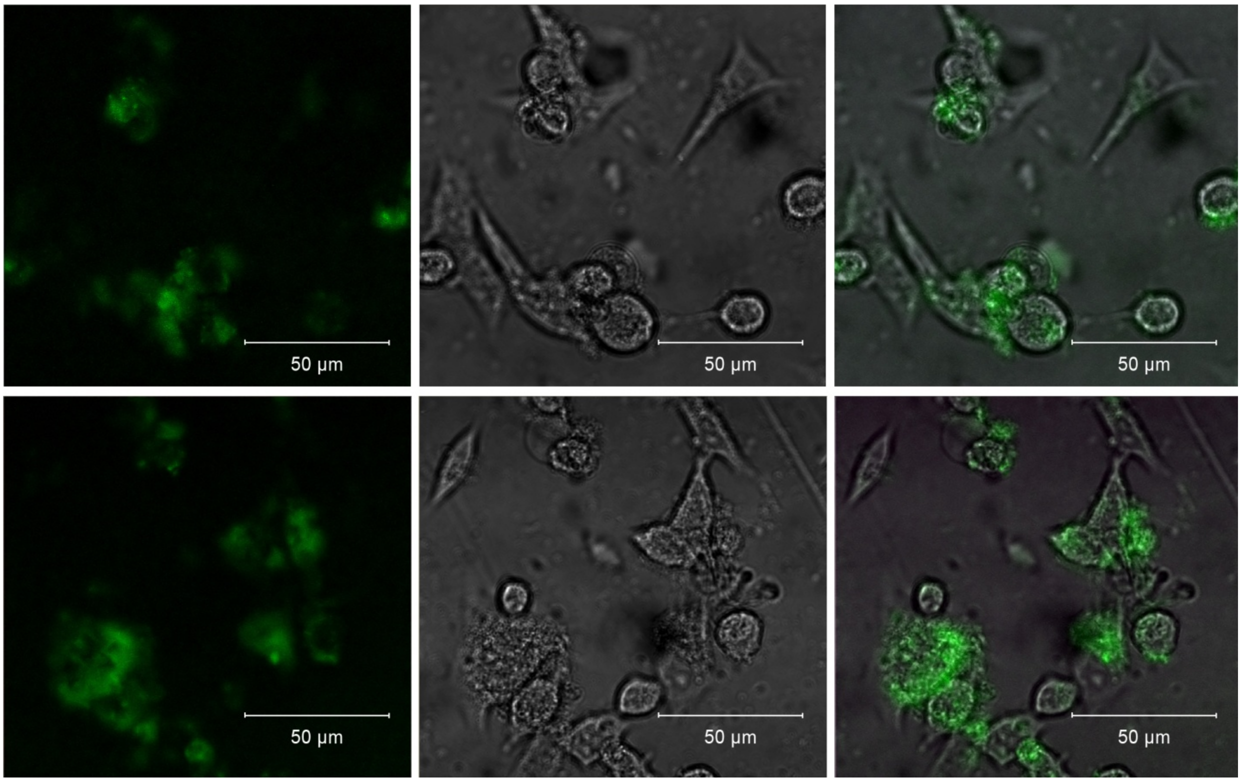

Figure S22: MCF-7S cells stained by O-dots (125 μg/mL) without fixation. Left to right: fluorescence microscopy image (488 nm excitation), bright-field microscopy image, and overlay.

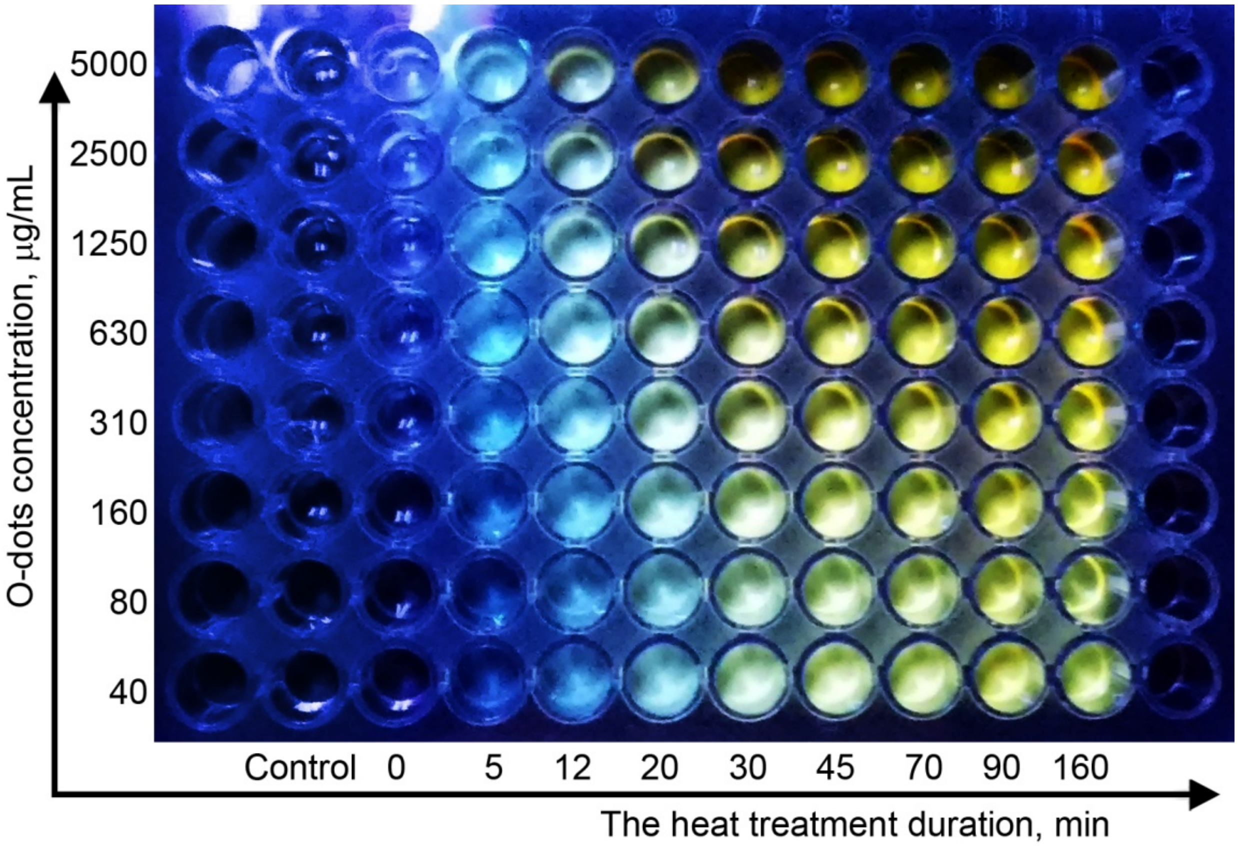

Figure S23: UV-illuminated multi-well plate with ST-cells supplemented with O-dots obtained by heat treatment of urea:citric acid mixture (5:1 mol) at 160 °C for 0–160 min.
